# Assessment of the Frequency and Variety of Persistent Symptoms Among Patients With COVID-19: A Systematic Review

**DOI:** 10.1001/jamanetworkopen.2021.11417

**Published:** 2021-05-26

**Authors:** Tahmina Nasserie, Michael Hittle, Steven N. Goodman

**Affiliations:** 1 Department of Epidemiology and Population Health, Stanford University, Stanford, California

## Abstract

**Importance:**

Infection with COVID-19 has been associated with long-term symptoms, but the frequency, variety, and severity of these complications are not well understood. Many published commentaries have proposed plans for pandemic control that are primarily based on mortality rates among older individuals without considering long-term morbidity among individuals of all ages. Reliable estimates of such morbidity are important for patient care, prognosis, and development of public health policy.

**Objective:**

To conduct a systematic review of studies examining the frequency and variety of persistent symptoms after COVID-19 infection.

**Evidence Review:**

A search of PubMed and Web of Science was conducted to identify studies published from January 1, 2020, to March 11, 2021, that examined persistent symptoms after COVID-19 infection. Persistent symptoms were defined as those persisting for at least 60 days after diagnosis, symptom onset, or hospitalization or at least 30 days after recovery from the acute illness or hospital discharge. Search terms included *COVID-19*, *SARS-CoV-2*, *coronavirus*, *2019-nCoV*, *long-term*, *after recovery*, *long-haul*, *persistent*, *outcome*, *symptom*, *follow-up*, and *longitudinal*. All English-language articles that presented primary data from cohort studies that reported the prevalence of persistent symptoms among individuals with SARS-CoV-2 infection and that had clearly defined and sufficient follow-up were included. Case reports, case series, and studies that described symptoms only at the time of infection and/or hospitalization were excluded. A structured framework was applied to appraise study quality.

**Findings:**

A total of 1974 records were identified; of those, 1247 article titles and abstracts were screened. After removal of duplicates and exclusions, 92 full-text articles were assessed for eligibility; 47 studies were deemed eligible, and 45 studies reporting 84 clinical signs or symptoms were included in the systematic review. Of 9751 total participants, 5266 (54.0%) were male; 30 of 45 studies reported mean or median ages younger than 60 years. Among 16 studies, most of which comprised participants who were previously hospitalized, the median proportion of individuals experiencing at least 1 persistent symptom was 72.5% (interquartile range [IQR], 55.0%-80.0%). Individual symptoms occurring most frequently included shortness of breath or dyspnea (26 studies; median frequency, 36.0%; IQR, 27.6%-50.0%), fatigue or exhaustion (25 studies; median frequency, 40.0%; IQR, 31.0%-57.0%), and sleep disorders or insomnia (8 studies; median 29.4%, IQR, 24.4%-33.0%). There were wide variations in the design and quality of the studies, which had implications for interpretation and often limited direct comparability and combinability. Major design differences included patient populations, definitions of time zero (ie, the beginning of the follow-up interval), follow-up lengths, and outcome definitions, including definitions of illness severity.

**Conclusions and Relevance:**

This systematic review found that COVID-19 symptoms commonly persisted beyond the acute phase of infection, with implications for health-associated functioning and quality of life. Current studies of symptom persistence are highly heterogeneous, and future studies need longer follow-up, improved quality, and more standardized designs to reliably quantify risks.

## Key Points

**Question:**

What are the frequency and variety of persistent symptoms after COVID-19 infection?

**Findings:**

In this systematic review of 45 studies including 9751 participants with COVID-19, the median proportion of individuals who experienced at least 1 persistent symptom was 73%; symptoms occurring most frequently included shortness of breath or dyspnea, fatigue or exhaustion, and sleep disorders or insomnia. However, the studies were highly heterogeneous and needed longer follow-up and more standardized designs.

**Meaning:**

This systematic review found that COVID-19 symptoms commonly persisted beyond the acute phase of infection, with implications for health-associated functioning and quality of life; however, methodological improvements are needed to reliably quantify these risks.

## Teaser

This systematic review uses data from cohort studies to examine the frequency, variety, and severity of persistent symptoms among individuals with previous COVID-19 infection.

## Introduction

The COVID-19 pandemic continues to spread, with the global case count and number of deaths estimated at 154 million and 3.2 million, respectively, as of May 5th, 2021. Other coronaviruses, such as those associated with severe acute respiratory syndrome and Middle East respiratory syndrome, have been associated with long-term complications after recovery.^1,2^

Health care professionals and patients have reported symptoms long after recovery from the acute phase of COVID-19 infection.^3,4^ The Centers for Disease Control and Prevention has stated that COVID-19 has consequences for many organ systems.^5^ Recently published commentaries have reported the prevalence of long-term outcomes across a range of studies, albeit with minimal critical scrutiny.^6,7^ Most studies of COVID-19 risks have focused on mortality, which is highest among older populations, and have omitted or minimized the disease burden associated with persistent or long-term morbidity among individuals of all ages. Reliable estimates of such morbidity are important for individual care, prognosis, and development of public health policy.

The primary objective of the present study was to systematically review existing literature examining the frequency and nature of persistent COVID-19 symptoms. A secondary objective was to systematically assess the design features of these studies to assess the reliability, comparability, and combinability of their outcome estimates and to improve the future evidence base for understanding the prevalence of long-term COVID-19 outcomes.

## Methods

This study followed the relevant sections of the Meta-analysis of Observational Studies in Epidemiology (MOOSE) reporting guideline for systematic reviews.

### Search Strategy and Selection Criteria

We performed a systematic search of PubMed and Web of Science for articles published between January 1, 2020, and March 11, 2021, to identify studies that assessed the prevalence of persistent symptoms among individuals with SARS-CoV-2 infection. We used the term *persistent* rather than *long-term* because the large majority of patients were assessed less than 100 days after diagnosis, symptom onset, hospital admission, or hospital discharge or less than 50 days after recovery from the acute illness. Search terms included *COVID-19*, *SARS-CoV-2*, *coronavirus*, *2019-nCoV*, *long-term*, *after recovery*, *long-haul*, *persistent*, *outcome*, *symptom*, *follow-up*, and *longitudinal*. The full search strategy is provided in eTable 1 in the Supplement.

Articles were considered relevant and eligible for inclusion if they (1) were written in the English language; (2) were cohort studies that reported the prevalence of persistent symptoms among individuals with SARS-CoV-2 infection; and (3) had clearly defined and sufficient follow-up. Studies that defined time zero (ie, the beginning of the follow-up interval) as symptom onset, COVID-19 diagnosis, or hospitalization owing to infection had to include a minimum of 2 months of follow-up; studies that defined time zero as recovery from the acute illness or hospital discharge had to include a minimum of 1 month of follow-up. We excluded case reports, case series, and articles that described symptoms only at the time of infection and/or hospitalization. Study quality was assessed, but studies were not excluded based on quality criteria.

### Identification of Studies

In the screening step, 1 of 2 authors (T.N. or M.H.) examined the titles and abstracts of articles using inclusion and exclusion criteria. In the eligibility step, 2 authors (T.N. and M.H.) examined the full text of each article to confirm that it met eligibility criteria. Disagreements were resolved by discussion between the 2 authors and involvement of a third author (S.G.) when necessary.

### Data Extraction and Quality Assessment

Two authors (T.N. and M.H.) independently extracted data from each article. Data extracted included study and patient characteristics, selection criteria, length of follow-up, and outcome measurements (Table 1).^8,9,10,11,12,13,14,15,16,17,18,19,20,21,22,23,24,25,26,27,28,29,30,31,32,33,34,35,36,37,38,39,40,41,42,43,44,45,46,47,48,49,50,51,52^ We used 6 quality criteria based on the National Institutes of Health Quality Assessment Tool for Observational and Cohort Studies^53^ to assess study design or features most likely to bias frequency estimates. Criteria comprised (1) prospective cohort (score range of 0-1, with 0 indicating no and 1 indicating yes), (2) representativeness (score range of 0-1, with 0 indicating sampling strategy unclear or nonconsecutive enrollees and 1 indicating patients were randomly selected or all eligible patients were included), (3) baseline severity of illness reported (score range of 0-1, with 0 indicating not reported and 1 indicating reported), (4) attrition (score range of 0-3, with 0 indicating not reported or attrition ≥30%, 1 indicating attrition of 20%-29%, 2 indicating attrition of 10%-19%, and 3 indicating attrition <10%), (5) repeated outcome measurements during study period (score range of 0-1, with 0 indicating outcomes were measured once and 1 indicating outcomes were measured more than once), and (6) established outcome scales to measure symptom prevalence (score range of 0-2, with 0 indicating no use, 1 indicating some use, and 2 indicating use for most outcomes) (Table 2).

**Table 1.** Evidence Table

Source	Country	Cohort type	Illness severity, mean (SD)^a^	Participant characteristics		Participants hospitalized, No./total No. in final sample (%)	Participants in ICU, No./total No. hospitalized (%)	Participant retention, No. in final sample/total No. eligible (%)	Outcome measurements
				Age, mean (SD), y	Male sex, No./total No. in final sample (%)				
Akter et al,^8^ 2020	Bangladesh	Nonconcurrent single-arm	NR	NR	558/734 (76.0)	734/734 (100)	NR	734/NR (NR)	Medical records
Arnold et al,^9^ 2020	United Kingdom	Concurrent single-arm	1.16 (0.37)	Mean (range), 47 (32-61)	68/110 (61.8)	110/110 (100)	NR	110/131 (84.0)	Radiography (chest abnormalities); blood sample (laboratory assessments); SF-36 (QOL); self-report (other outcomes)
Carfi et al,^10^ 2020	Italy	Concurrent single-arm	NR	56.5 (14.6)	90/143 (62.9)	143/143 (100)	18/143 (12.6)	143/157 (91.1)	EQ-VAS (QOL); patient survey (other outcomes)
Carvalho-Schneider et al,^11^ 2021	France	Concurrent, single-arm	1.22 (0.41)	49 (15)	62/130 (47.7)	46/130 (35.4)	0	130/174 (74.7)	mMRC Dyspnea Scale (dyspnea); 10-point analog scale (chest pain, anosmia, and ageusia)
Chen et al,^12^ 2020	China	Concurrent single-arm	1.09 (0.29)	47.2 (13.0)	186/361 (51.5)	361/361 (100)	NR	361/503 (71.8)	SF-36 (QOL)
Chiesa-Estomba et al,^13^ 2020	Spain	Concurrent single-arm	1.00 (0)	41 (13)	274/751 (36.5)	NR	NR	751/1222 (61.5)	QOD-NS (olfactory function)
Chopra et al,^14^ 2020	US	Concurrent single-arm	NR	Median (IQR), 62 (50-72)	253/488 (51.8)	488/488 (100)	NR	488/1167 (41.8)	Self-report
D’Cruz et al,^15^ 2021	United Kingdom	Concurrent single-arm	2.34 (0.22)	58.7 (14.4)	74/119 (62.2)	119/119 (100)	41/119 (34.5)	119/143 (83.2)	mMRC Dyspnea Scale (dyspnea); PHQ-9 (depression); TSQ (trauma); GAD-7 (anxiety); 6-CIT (cognitive impairment); CT scan (organ function); 4MGS (gait speed); 1-min sit-to-stand test (mobility)
Daher et al,^16^ 2020	Germany	Concurrent single-arm	NR	64 (3)	22/33 (66.7)	33/33 (100)	NR	33/NR (NR)	PHQ-9 (depression); GAD-7 (anxiety); EQ-5D-5L (QOL); 6-min walk test (mobility); blood sample (laboratory assessments); electrocardiography and CT scan (organ function)
de Graaf et al,^17^ 2021	Netherlands	Concurrent single-arm	NR	60.8 (13.0)	51/81 (63.0)	81/81 (100)	33/81 (40.7)	81/98 (82.7)	CT scan (organ function); pulmonary function tests; GAD-7 (anxiety); PHQ-9 (depression); PCL-5 (PTSD); CFQ-25 (cognitive impairment); IQCODE-N (cognitive impairment in older patients); NYHA (dyspnea)
Garrigues et al,^18^ 2020	France	Concurrent single-arm	NR	63.2 (15.7)	75/120 (62.5)	120/120 (100)	24/120 (20.0)	120/204 (58.8)	mMRC Dyspnea Scale (dyspnea); EQ-5D-5L (QOL); self-report (other outcomes)
Gherlone et al,^19^ 2021	Italy	Concurrent single-arm	2.25 (0.43)	Median (IQR), 62.5 (53.9-74.1)	92/122 (75.4)	122/122 (100)	30/122 (24.6)	122/NR (NR)	Extraoral and intraoral physical examinations (facial abnormalities)
Gonzalez et al,^20^ 2021	Spain	Concurrent single-arm	3.00 (0)	Median (IQR), 60 (48-65)	46/62 (74.2)	62/62 (100)	62/62 (100)	62/75 (82.7)	SF-12 (QOL); HADS (depression); CT scan (organ function); mMRC Dyspnea Scale (dyspnea); pulmonary function test
Halpin et al,^21^ 2021	United Kingdom	Concurrent single-arm	NR	Hospital ward: median (range), 70.5 (20.0-93.0) ICU: median (range), 58.5 (34.0-84.0)	54/100 (54.0)	100/100 (100)	32/100 (32.0)	100/158 (63.3)	EQ-5D-5L (QOL); telephone screening tool (other outcomes)
Huang et al,^22^ 2021	China	Concurrent single-arm	1.79 (0.50)	Median (IQR), 57 (47-65)	897/1733 (51.8)	1733/1733 (100)	76/1733 (4.4)	1733/2142 (80.9)	mMRC Dyspnea Scale (dyspnea); EQ-5D-5L (QOL, anxiety, and depression); EQ-VAS (QOL); blood sample (lab assessment); CT scan (organ function); 6-min walk test (mobility)
Jacobs et al,^23^ 2020	US	Concurrent single-arm	1.13 (0.33)	Median (IQR), 57 (48-68)	112/183 (61.2)	183/183 (100)	NR	183/351 (52.1)	PROMIS Global-10 (all outcomes)
Lechien et al,^24^ 2020	Belgium	Concurrent single-arm	1.00 (0)	46.2 (11.2)	29/88 (33.0)	0	0	88/95 (92.6)	SNOT-22 (sinonasal outcomes); QOD-NS (olfactory function); NHANES (olfactory and gustatory function); 16-item Sniffin-Sticks identification test (psychosocial olfactory evaluation)
Lerum et al,^25^ 2020	Norway	Concurrent single-arm	NR	Median (IQR), 59 (49-72)	54/103 (52.4)	103/103 (100)	15/103 (14.6)	103/NR (NR)	mMRC Dyspnea Scale (dyspnea); EQ-5D-5L (QOL); chest CT scan (organ function)
Liang et al,^26^ 2020	China	Concurrent single-arm	2.09 (0.29)	41.3 (13.8)	21/76 (27.6)	76/76 (100)	7/76 (9.2)	76/134 (56.7)	Spirometry (pulmonary function); CT scan (organ function); blood sample (laboratory assessments)
Lu et al,^27^ 2020	China	Concurrent double-arm	1.24 (0.47)	44.1 (16.0)	34/60 (56.7)	60/60 (100)	NR	60/155 (38.7)	MRI scan (cerebral activity); self-report (other outcomes)
Mandal et al,^28^ 2020	United Kingdom	Concurrent single-arm	1.31 (0.46)	59.9 (16.1)	238/384 (62.0)	384/384 (100)	56/384 (14.6)	384/878 (43.7)	Radiography (chest abnormalities); blood sample (laboratory assessments); PHQ-2 (depression); self-report (other outcomes)
Mazza et al,^29^ 2021	Italy	Concurrent single-arm	NR	58.5 (12.8)	149/226 (65.9)	177/226 (78.3)	NR	226/402 (56.2)	IES-R (distress); PCL-5 (PTSD); ZSDS (depression); BDI-13 (depression); STAI-Y (anxiety); WHIIRS (insomnia); OCI (obsessive-compulsive disorder); BACS (cognitive function); clinical records (inflammatory markers)
Mendez et al,^30^ 2021	Spain	Nonconcurrent single-arm	NR	Median (IQR), 57 (49-67)	105/179 (58.7)	179/179 (100)	34/179 (19.0)	179/216 (82.9)	SF-12 (QOL); SCIP (verbal memory); ANT (verbal fluency); WAIS-III (working memory); GAD-7 (anxiety); PHQ-2 (depression); DTS (PTSD)
Moreno-Perez et al,^31^ 2021	Spain	Concurrent single-arm	1.66 (0.47)	Median (IQR), 62 (53-72)	146/277 (52.7)	277/277 (100)	24/277 (8.7)	277/326 (85.0)	EQ-VAS (QOL); radiography (chest abnormalities); blood sample (laboratory assessments); pulmonary function test
Munro et al,^32^ 2020	United Kingdom	Concurrent single-arm	NR	NR	NR	121/121 (100)	2/121 (1.7)	121/NR (NR)	General questionnaire
Nguyen et al,^33^ 2021	France	Concurrent single-arm	NR	Median (IQR), 36 (27-48)	56/125 (44.8)	0	0	125/200 (62.5)	Self-report
Poncet-Megemont et al,^34^ 2020	France	Nonconcurrent single-arm	1.45 (0.57)	48.5 (15.3)	52/139 (37.4)	63/139 (45.3)	6/139 (4.3)	139/161 (86.3)	Self-report
Puntmann et al,^35^ 2020	Germany	Concurrent double-arm	1.15 (0.70)	49 (14)	53/100 (53.0)	33/100 (33.0)	NR	100/NR (NR)	MRI scan (cardiac activity); self-report (other outcomes)
Qu et al,^36^ 2021	China	Concurrent single-arm	1.09 (0.29)	Median (IQR), 47.5 (37.0-57.0)	270/540 (50.0)	540/540 (100)	NR	540/573 (94.2)	SF-36 (QOL); self-report (other outcomes)
Raman et al,^37^ 2021	United Kingdom	Concurrent double-arm	NR	55.4 (13.2)	34/58 (58.6)	58/58 (100)	21/58 (36.2)	58/NR (NR)	MRI scan (organ activity); spirometry (lung function); 6-min walk test (mobility); PHQ-9 (depression); GAD-7 (anxiety); MoCA (cognitive function); mMRC Dyspnea Scale (dyspnea); FSS (fatigue); SF-36 (QOL)
Rosales-Castillo et al,^38^ 2021	Spain	Nonconcurrent single-arm	NR	60.2 (15.1)	66/118 (55.9)	118/118 (100)	9/118 (7.6)	118/NR (NR)	Self-report
Shah et al,^39^ 2020	Canada	Concurrent single-arm	NR	Median (IQR), 67 (54-74)	41/60 (68.3)	60/60 (100)	NR	60/82 (73.2)	Detailed pulmonary function test; 6-min walk test (mobility); CT scan (organ function); UCSD SOBQ (dyspnea)
Sonnweber et al,^40^ 2020	Austria	Concurrent single-arm	1.72 (0.80)	57 (14)	73/133 (54.9)	99/133 (74.4)	29/133 (21.8)	133/190 (70.0)	mMRC Dyspnea Scale (dyspnea); spirometry and blood plethysmography (pulmonary function); chest CT scan (organ function); blood sample (laboratory assessments); transthoracic echocardiography (cardiac function)
Sonnweber et al,^41^ 2020	Austria	Concurrent single-arm	1.66 (0.75)	58 (14)	65/109 (59.6)	87/109 (79.8)	18/109 (16.5)	109/186 (58.6)	6-min walk test (mobility); CT scan (lung function); blood sample (laboratory assessments), questionnaire (other outcomes)
Sykes et al,^42^ 2021	England	Concurrent single-arm	NR	59.6 (14.0)	88/134 (65.7)	134/134 (100)	27/134 (20.1)	134/190 (70.5)	Radiography (chest abnormalities); mMRC Dyspnea Scale (dyspnea); EQ-5D-5L (QOL); direct questioning (other outcomes)
Taboada et al,^43^ 2021	Spain	Concurrent single-arm	3.00 (0)	65.5 (10.4)	59/91 (64.8)	91/91 (100)	91/91 (100)	91/92 (98.9)	EQ-5D-5L (QOL); PCFS (functional status)
Tomasoni et al,^44^ 2021	Italy	Concurrent single-arm	NR	Median (range), 55 (43-65)	77/105 (73.3)	105/105 (100)	NR	105/NR (NR)	HADS (anxiety and depression); MMSE (cognitive disorders)
Townsend et al,^45^ 2020	Ireland	Concurrent single-arm	NR	49.15 (15.00)	59/128 (46.1)	71/128 (55.5)	18/128 (14.1)	128/223 (57.4)	CFQ-11 (fatigue)
Ugurlu et al,^46^ 2021	Turkey	Concurrent single-arm	NR	41.2 (14.6)	19/42 (45.2)	42/42 (100)	0	42/42 (100)	BSIT (olfactory function)
Vaira et al,^47^ 2020	Italy	Concurrent single-arm	NR	51.2 (8.8)	68/138 (49.3)	32/138 (23.2)	0	138/146 (94.5)	Self-administered olfactory and gustatory psychosocial tests (anosmia and ageusia/dysgeusia for outpatients); CCCRC Orthonasal Olfaction Test (anosmia and ageusia/dysgeusia for inpatients)
van den Borst et al,^48^ 2020	Netherlands	Concurrent single-arm	1.53 (0.76)	59 (14)	74/124 (59.7)	97/124 (78.2)	20/97 (20.6)	124/197 (62.9)	Resting pulse-oximetry and spirometry (pulmonary functioning); mMRC Dyspnea Scale (dyspnea); CT scan and radiography (chest function); CFS (frailty); HADS (anxiety and depression); TICS and CFQ (cognitive function); PCL-5 and IES-R (PTSD); SF-36 (QOL); blood sample (laboratory assessments)
Weerahandi et al,^49^ 2021	US	Concurrent single-arm	2.00 (0)	Median (IQR), 62 (50-67)	95/152 (62.5)	152/152 (100)	70/152 (46.1)	152/397 (38.3)	PROMIS Global-10 (all outcomes)
Wong et al,^50^ 2020	Canada	Concurrent single-arm	NR	62 (16)	50/78 (64.1)	78/78 (100)	NR	78/96 (81.3)	EQ-5D-5L (QOL); UCSD Frailty Index (frailty); UCSD SOBQ (shortness of breath); Pittsburgh Sleep Quality Index (sleep quality); PHQ-9 (depression)
Xiong et al,^51^ 2021	China	Nonconcurrent double-arm	1.44 (0.59)	Median (IQR), 52 (95-102)	245/538 (45.5)	538/538 (100)	NR	538/706 (76.2)	Medical records
Zhao et al,^52^ 2020	China	Nonconcurrent single-arm	1.07 (0.26)	47.7 (15.5)	32/55 (58.2)	55/55 (100)	0	55/73 (75.3)	Medical records; CT scan (chest function); spirometry (pulmonary function); self-report (other outcomes)

Abbreviations: 4MGS, 4-m gait speed; ANT, Animal Naming Test; BACS, Brief Assessment of Cognition in Schizophrenia; BDI-13, 13-item Beck Depression Inventory; BSIT, Brief Smell Identification Test; CCCRC, Connecticut Chemosensory Clinical Research Center; CFQ, Cognitive Failure Questionnaire; CFQ-11, 11-item Chalder Fatigue Scale; CFQ-25, 25-item Cognitive Failure Questionnaire; CFS, Clinical Frailty Score; CT, computed tomography; DTS, Davidson Trauma Scale; EQ-5D-5L, EuroQol 5-dimension 5-level scale; EQ-VAS, EuroQol Visual Analog Scale; FSS, Fatigue Severity Scale; GAD-7, 7-item General Anxiety Disorder Scale; HADS, Hospital Anxiety and Depression Scale; ICU, intensive care unit; IES-R, Impact of Event Scale–Revised; IQCODE-N, Informant Questionnaire on Cognitive Decline in the Elderly–Netherlands; IQR, interquartile range; mMRC, modified Medical Research Council; MMSE, Mini-Mental State Examination; MoCA, Montreal Cognitive Assessment; MRI, magnetic resonance imaging; NHANES, National Health and Nutrition Examination Survey; NR, not reported; NYHA, New York Heart Association; OCI, Obsessive-Compulsive Inventory; PCFS, Post–COVID-19 Functional Status Scale; PCL-5, Posttraumatic Stress Disorder Checklist for DSM-5; PHQ-2, 2-item Patient Health Questionnaire; PHQ-9, 9-item Patient Health Questionnaire; PROMIS Global-10, 10-item Patient-Reported Outcomes Measurement Information System Global Health instrument; PTSD, posttraumatic stress disorder; QOD-NS, Questionnaire of Olfactory Disorders–Negative Statements; QOL, quality of life; SCIP, Screen for Cognitive Impairment in Psychiatry; SF-12, 12-Item Short Form Survey; SF-36, 36-Item Short Form Survey; SNOT-22, Sino-Nasal Outcome Test; 6-CIT, six-item Cognitive Impairment Test; STAI-Y, State-Trait Anxiety Inventory–Form Y; TICS, Telephone Interview for Cognitive Status; TSQ, Trauma Screening Questionnaire; UCSD SOBQ, University of California San Diego Shortness of Breath Questionnaire; WAIS-III, Wechsler Adult Intelligence Scale, Third Edition; WHIIRS, Women’s Health Initiative Insomnia Rating Scale; ZSDS, Zung Self-rating Depression Scale.

^a^ Disease severity at baseline was calculated as a weighted mean (the sum of all severity scores multiplied by the proportion of patients with that score). Severity scores were 0 (asymptomatic), 1 (mild or moderate), 2 (severe), and 3 (critical).

**Table 2.** Quality Assessment of Included Studies

Source	Prospective cohort (0 or 1)^a^	Representativeness (0 or 1)^b^	Baseline severity reported (0 or 1)^c^	Attrition (0, 1, 2, or 3)^d^	Repeated outcome measurements (0 or 1)^e^	Established outcome scales (0, 1, or 2)^f^
Akter et al,^8^ 2020	0	0	0	0	0	0
Arnold et al,^9^ 2020	1	1	1	2	0	1
Carfi et al,^10^ 2020	1	1	0	3	0	1
Carvalho-Schneider et al,^11^ 2021	1	1	1	1	1	1
Chen et al,^12^ 2020	1	0	1	1	0	2
Chiesa-Estomba et al,^13^ 2020	1	0	1	0	0	1
Chopra et al,^14^ 2020	1	1	0	0	0	0
D’Cruz et al,^15^ 2021	1	1	1	2	0	2
Daher et al,^16^ 2020	1	1	0	0	0	1
de Graaf et al,^17^ 2021	1	1	0	2	0	2
Garrigues et al,^18^ 2020	1	1	0	0	0	1
Gherlone et al,^19^ 2021	1	1	1	0	0	0
Gonzalez et al,^20^ 2021	1	1	1	2	0	1
Halpin et al,^21^ 2021	1	1	0	0	0	1
Huang et al,^22^ 2021	1	1	1	2	0	2
Jacobs et al,^23^ 2020	1	1	1	0	1	2
Lechien et al,^24^ 2020	1	0	1	3	1	2
Lerum et al,^25^ 2020	1	1	0	0	0	2
Liang et al,^26^ 2020	1	0	1	0	0	1
Lu et al,^27^ 2020	1	1	1	0	0	1
Mandal et al,^28^ 2020	1	1	1	0	0	1
Mazza et al,^29^ 2021	1	0	0	0	1	2
Mendez et al,^30^ 2021	0	0	0	2	0	2
Moreno-Perez et al,^31^ 2021	1	1	1	2	0	1
Munro et al,^32^ 2020	1	0	0	0	0	0
Nguyen et al,^33^ 2021	1	1	0	0	0	0
Poncet-Megemont et al,^34^ 2020	0	1	1	2	0	0
Puntmann et al,^35^ 2020	1	1	1	0	0	0
Qu et al,^36^ 2021	1	0	1	3	0	1
Raman et al,^37^ 2021	1	1	0	0	0	2
Rosales-Castillo et al,^38^ 2021	1	0	0	0	0	0
Shah et al,^39^ 2020	1	1	0	1	0	2
Sonnweber et al,^40^ 2020	1	0	1	0	1	2
Sonnweber et al,^41^ 2020	1	0	1	0	0	2
Sykes et al,^42^ 2021	1	1	0	1	0	1
Taboada et al,^43^ 2021	1	1	1	3	0	1
Tomasoni et al,^44^ 2021	1	0	0	0	0	2
Townsend et al,^45^ 2020	1	1	0	0	0	2
Ugurlu et al,^46^ 2021	1	1	0	3	0	2
Vaira et al,^47^ 2020	1	0	0	3	1	2
van den Borst et al,^48^ 2020	1	1	0	0	0	2
Weerahandi et al,^49^ 2021	1	1	1	0	0	2
Wong et al,^50^ 2020	1	1	0	2	0	2
Xiong et al,^51^ 2021	0	1	1	1	0	0
Zhao et al,^52^ 2020	0	1	1	1	0	0

^a^ Score of 0 indicates no (5 studies) and 1 indicates yes (40 studies).

^b^ Score of 0 indicates sampling strategy unclear or nonconsecutive enrollees (14 studies) and 1 indicates patients randomly selected or all eligible patients included (31 studies).

^c^ Score of 0 indicates not reported (22 studies) and 1 indicates reported (23 studies).

^d^ Score of 0 indicates not reported or attrition of 30% or higher (24 studies), 1 indicates attrition of 20% to 29% (6 studies), 2 indicates attrition of 10% to 19% (9 studies), and 3 indicates attrition of less than 10% (6 studies).

^e^ Score of 0 indicates outcome measured once (39 studies) and 1 indicates outcome measured more than once (6 studies).

^f^ Score of 0 indicates no use of outcome scales (10 studies), 1 indicates some use of outcome scales (15 studies), and 2 indicates use of outcome scales for most outcomes (20 studies).

### Data Synthesis

#### Study Design and Reporting

We recorded the main design elements of each study and the ways in which data were reported. This information was used to develop methodological recommendations to reduce variation in design and improve uniformity and completeness of reporting in future research.

#### Persistent Symptoms

Persistent symptoms were defined as those persisting for at least 60 days after diagnosis, symptom onset, or hospital admission or at least 30 days after recovery from acute illness or hospital discharge. The range of persistent COVID-19 symptoms reported to date was identified and categorized. We recorded the percentage of individuals experiencing each outcome at the follow-up time specified in the studies. If outcomes were measured more than once during the follow-up period, we reported the percentage of individuals at the last follow-up time.

### Statistical Analysis

We used a descriptive approach to the analysis because the heterogeneity of study designs limited the combinability of most estimates. The median and interquartile range (IQR) were reported for outcomes with 5 or more estimates, and individual values were reported for outcomes with 4 or fewer estimates. We did not report 95% CIs for the reported percentages because they were not directly relevant to inferences and, in most cases, reported frequencies varied more by design than could be attributed to random error. Disease severity at baseline was calculated as a weighted mean (the sum of all severity scores multiplied by the proportion of patients with that score). Severity scores were 0 (asymptomatic), 1 (mild to moderate), 2 (severe), and 3 (critical).

**Figure 1.**
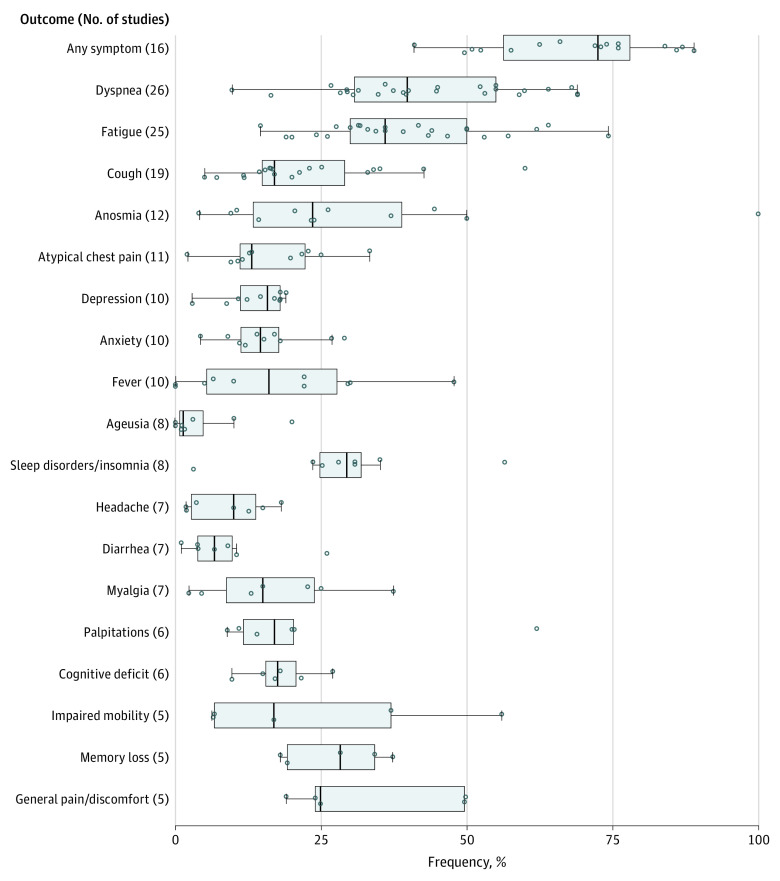
Reported Frequencies of Symptoms Examined by 5 or More Studies The horizontal bar extends from the first to the third quartile, the interquartile range (IQR). The whiskers extend from the upper and lower quartiles to the largest value within 1.5 IQRs of that quartile. The width of the box represents the IQR. The vertical bar represents the median value for the outcome. The circles represent point estimates from each study. Circles beyond the whiskers are considered outliers. Values for anosmia (loss of smell) and ageusia (loss of taste) represent frequency of loss if that loss began during acute stage of infection among studies with available data. Therefore, 7 studies reporting anosmia and 5 studies reporting ageusia were excluded from the figure.

Risk estimates for the outcomes examined in 10 or more studies and for quality-of-life measures are summarized in the text, and outcomes examined in 5 or more studies are displayed graphically (Figure 1). When possible, we explored whether differences in study design could have been associated with variation in estimates between studies.

## Results

The most salient feature of included studies was heterogeneity in design, even in single dimensions (eg, follow-up period or symptom measurement). In this section, design features are summarized followed by quantitative results.

### Design Features

#### Study Characteristics

A total of 1974 records were identified; of those, 1247 article titles and abstracts were screened. After removal of duplicates and exclusions, 92 full-text articles were assessed for eligibility; 47 studies were deemed eligible, and 45 studies (including 9751 participants reporting 84 clinical signs or symptoms) were included in the systematic review (eFigure and eTable 2 in the Supplement).^8,9,10,11,12,13,14,15,16,17,18,19,20,21,22,23,24,25,26,27,28,29,30,31,32,33,34,35,36,37,38,39,40,41,42,43,44,45,46,47,48,49,50,51,52^ Overall, 7 studies were conducted in China^12,22,26,27,36,51,52^; 6 each in the United Kingdom^9,15,21,28,32,37^ and Spain^13,20,30,31,36,43^; 5 in Italy^10,19,29,47,51^; 4 in France^11,18,33,34^; 3 in the US^14,23,49^; 2 each in Germany,^16,35^ Canada,^39,50^ the Netherlands,^17,48^ and Austria^40,41^; and 1 each in Ireland,^45^ Norway,^25^ Turkey,^46^ Belgium,^24^ England,^42^ and Bangladesh.^8^ Among the 45 studies, 33^8,9,10,11,12,13,14,15,18,19,21,22,23,25,28,29,30,31,32,33,34,35,36,38,40,41,42,44,45,47,48,49,51^ included a final sample of at least 100 individuals (median number of participants, 122.0; IQR, 89.5-181.0) (eTable 3 in the Supplement).

#### Patient Selection Criteria

Thirty-three studies recruited only inpatients; 10 studies^11,13,29,34,35,40,41,45,47,48^ included a combination of outpatients and inpatients, with the proportion of inpatients ranging from 23.0% to 80.0% (Table 1), and 2 studies^24,33^ included only outpatients. Three studies excluded patients who were unable or unwilling to receive a magnetic resonance imaging scan.^27,35,37^ Fourteen studies^8,14,16,19,23,25,27,29,32,35,37,38,44,45^ did not report reasons for nonparticipation and/or the corresponding number of individuals excluded (eTable 4 in the Supplement).

#### Patient Characteristics

Among 9751 total participants, 5266 (54.0%) were male; 30 studies^9,10,11,12,13,15,22,23,24,25,26,27,28,29,30,33,34,35,36,37,40,41,42,44,45,46,47,48,51,52^ reported mean or median ages younger than 60 years, and 14 studies^9,11,12,13,24,26,27,33,34,35,36,45,46,52^ reported mean or median ages of 50 years or younger (Table 1). Twenty-four studies^9,11,12,13,15,19,20,22,23,24,26,27,28,31,34,35,36,40,41,43,48,49,51,52^ reported the baseline severity of COVID-19 illness, which varied substantially, even among hospitalized patients. Of those, 19 studies^9,11,12,15,19,22,23,26,27,28,31,34,35,36,40,41,48,51,52^ included patients with 2 or more symptom severity levels (asymptomatic, mild to moderate, severe, or critical). In the remaining 5 studies, all patients had mild to moderate (n = 2),^13,24^ severe (n = 1),^49^ or critical (n = 2)^20,43^ symptom severity. Forty studies^8,9,10,11,12,13,14,15,16,17,18,19,20,21,22,23,24,25,26,27,28,30,31,33,35,37,38,39,40,41,42,43,44,45,47,48,49,50,51,52^ reported the prevalence of underlying comorbidities in the study population. The most commonly reported comorbidities were diabetes (34 studies^8,10,13,14,15,16,17,18,19,20,22,23,24,25,26,27,28,30,31,33,35,37,38,39,40,41,42,43,47,48,49,50,51,52^; median frequency, 16.6%; IQR, 10.0%-23.0%) and hypertension (32 studies^9,10,13,14,16,17,18,19,20,21,22,23,24,25,26,27,28,30,31,33,35,37,38,39,40,41,42,43,48,49,51,52^; median frequency, 35.0%; IQR, 21.8%-41.0%) (eTable 3 in the Supplement).

#### Follow-up

**Figure 2.**
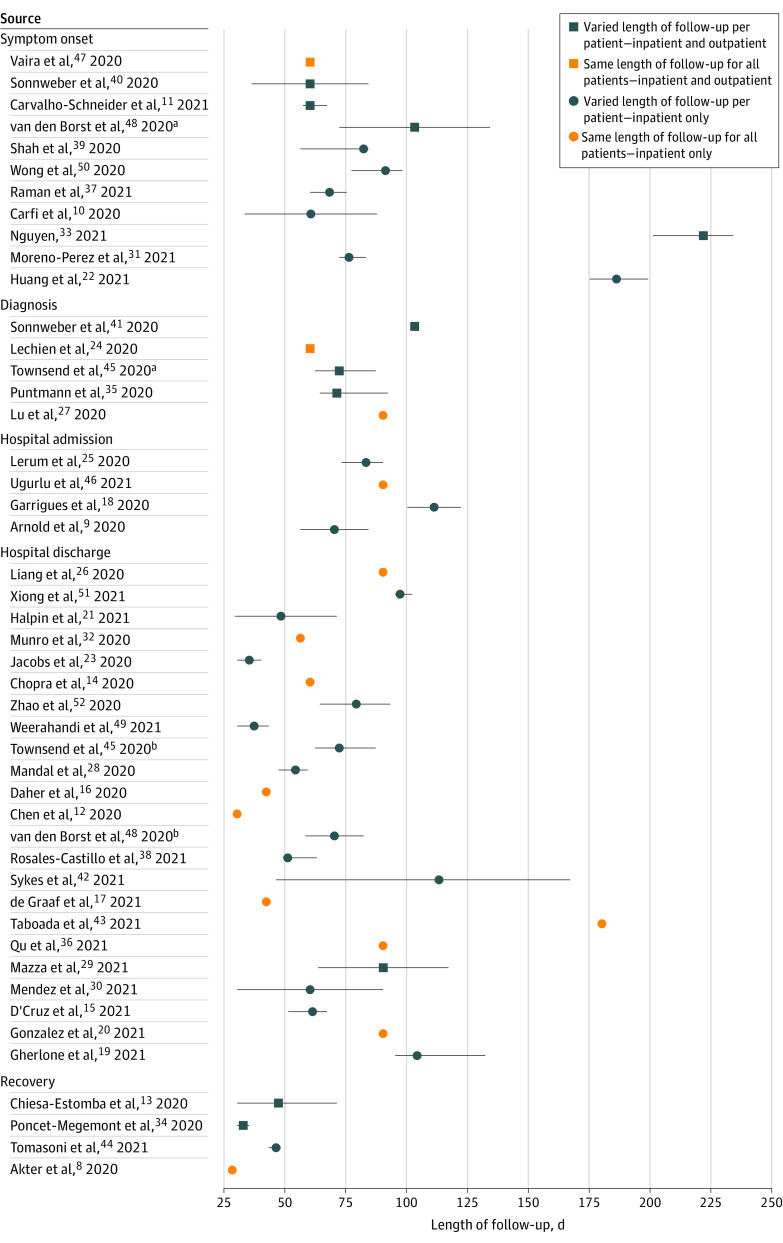
Overview of Time Zero Definitions and Follow-up Periods for Each Patient Across Included Studies The figure depicts heterogeneity in the definitions of time zero (symptom onset, diagnosis, hospital admission, hospital discharge, or recovery from the acute illness), patient care settings, and lengths and types of follow-up across studies. Patients were followed up from time zero until the end of follow-up, which either was consistent for all patients within a study or varied per patient depending on the date of the last medical examination. Summary statistics varied, with some studies reporting the mean (SD) of follow-up time and others reporting the median (IQR) or another nonparametric summary. Error bars indicate the minimum and maximum length of follow-up for individual patients. ^a^Outpatients only. ^b^Inpatients only.

Time zero definitions and lengths of follow-up varied substantially across studies, with very few studies using identical approaches to defining time zero, follow-up, and reporting. Time zero was defined as diagnosis or symptom onset in 16 studies,^10,11,22,24,27,31,33,35,37,39,40,41,45,47,48,50^ hospital admission in 4 studies,^9,18,25,46^ hospital discharge in 23 studies,^12,14,15,16,17,19,20,21,23,26,28,29,30,32,36,38,42,43,45,48,49,51,52^ and recovery from acute illness in 4 studies.^8,13,34,44^ Two studies used different time zero definitions for outpatients vs inpatients within the same study.^45,48^ Follow-up duration was similarly variable. Fourteen studies^8,12,14,16,17,20,24,26,27,32,36,43,46,47^ followed up all participants for a specified time. In the remaining studies,^9,10,11,13,15,18,19,21,22,23,25,28,29,30,31,33,34,35,37,38,39,40,41,42,44,45,48,49,50,51,52^ the end of follow-up and the duration of symptoms were determined by the date of the last medical examination. Summary statistics also varied, with some studies^10,13,18,21,23,33,34,39,40,48^ reporting the mean (SD) of follow-up time and others^8,9,11,12,14,15,16,17,19,20,22,24,25,26,27,28,29,30,31,32,35,36,37,38,41,42,43,44,45,46,47,49,50,51,52^ reporting the median (IQR) or another nonparametric summary. Figure 2 shows all of the combinations of time zero definitions, follow-up times, reporting summaries, and patient strata (with supporting data available in eTable 5 in the Supplement).

#### Outcomes Studied

The full list of outcomes is presented in eTable 6 in the Supplement. We included outcomes measuring quality of life and findings from radiography and cardiac magnetic resonance imaging. The included studies reported 84 signs or symptoms and 19 laboratory or imaging measurements. The most commonly examined symptoms were shortness of breath or dyspnea (26 studies^9,10,11,15,16,17,18,20,21,22,23,25,28,31,35,36,37,38,39,40,42,43,44,49,50,52^), fatigue or exhaustion (25 studies^9,10,11,15,16,18,20,21,22,23,26,27,28,31,35,36,37,38,42,43,44,45,48,51,52^), cough (19 studies^9,14,15,16,18,20,21,23,26,28,31,36,38,39,40,42,43,50,51^), depression and/or anxiety (16 studies^8,15,17,20,21,22,27,28,29,30,37,42,44,48,50,51^), anosmia or loss of smell (19 studies^9,13,16,18,19,20,22,23,24,27,33,34,38,40,42,43,44,46,47^), ageusia or loss of taste (13 studies^16,18,19,20,22,23,27,33,34,38,42,44,47^), and atypical chest pain (11 studies^9,10,11,16,17,18,23,35,42,43,51^).

#### Outcome Measurements

Most studies^8,9,10,12,13,14,15,16,17,18,19,20,21,22,25,26,27,28,30,31,32,33,34,35,36,37,38,39,41,42,43,44,45,46,48,49,50,51,52^ measured outcomes at a single follow-up time and reported the percentage of the study population that continued to experience the outcome at the end of follow-up. Thirty-five studies^9,10,11,12,13,15,16,17,18,20,21,22,23,24,25,26,27,28,29,30,31,36,37,39,40,41,42,43,44,45,46,47,48,49,50^ used standardized scales to measure some or all included outcomes. Quality of life measures were most commonly assessed using questionnaires, including the EuroQol 5-dimension 5-level questionnaire^54^ (10 studies^10,16,18,21,22,25,31,42,43,50^) and the 36-Item Short Form Survey^55^ (5 studies^9,12,36,37,48^). Other outcomes measured by standardized questionnaires included fatigue, dyspnea, and anxiety and/or depression, with variation in the instruments used across studies (Table 1).

#### Study Quality

Factors associated with the quality of evidence are presented in Table 2. The variable that was most representative of low study quality was attrition, which was reported in 36 of 45 studies^9,10,11,12,13,14,15,17,18,20,21,22,23,24,26,27,28,29,30,31,33,34,36,39,40,41,42,43,45,46,47,48,49,50,51,52^ (80.0%). In total, 24 studies^8,13,14,16,18,19,21,23,25,26,27,28,29,32,33,35,37,38,40,41,44,45,48,49^ (53.3%) either did not report retention or reported retention of 70.0% or less among patients from the initial eligible sample. Among studies that reported retention, the median was 74.0% (IQR, 60.0%-83.6%), with only 15 studies^9,10,15,17,20,22,24,30,31,34,36,43,46,47,50^ (33.3%) exceeding 80% retention and 6 studies^10,24,36,43,46,47^ (13.3%) exceeding 90% retention (Table 1). Most studies did not report the demographic characteristics of patients who declined participation. A total of 31 studies^9,10,11,14,15,16,17,18,19,20,21,22,23,25,27,28,31,33,34,35,37,39,42,43,45,46,48,49,50,51,52^ (68.9%) randomly selected patients or included all eligible patients. Other variables associated with study quality were the frequency of outcome measurements (with outcomes measured more than once in only 6 studies^11,23,24,29,40,47^) and the reporting of baseline illness severity (23 studies^8,9,11,12,13,15,19,20,22,23,24,26,27,28,31,34,35,36,40,41,43,49,51^). Twenty studies^12,15,17,22,23,24,25,29,30,37,39,40,41,44,45,46,47,48,49,50^ used standardized scales to measure most or all outcomes. Although we did not create a composite quality score because of the different implications of these dimensions for risk of bias, almost all studies were of moderate or low quality based only on retention, standardization, and representativeness criteria. Based on our findings, we formulated recommendations for improving quality and design in the domains of study population, recruitment strategy, follow-up, exposure measurement, outcomes of interest, outcome measurement, and results (Table 3).

**Table 3.** Methodological Recommendations for Future Studies of Persistent COVID-19 Symptoms

Category	Recommendations
Study population	Report underlying comorbidities (based on WHO^56^ and CDC^57^ guidelines), including hypertension, type 2 diabetes, obesity, chronic kidney disease, cancer, compromised immunity, COPD, heart conditions, and smoking
	Report prevalence of symptoms before COVID-19 infection
	Report severity of COVID-19 illness: asymptomatic or mild, moderate, severe, and critical using standard COVID-19 symptom severity scales (eg, WHO^56^)
	Report patient care settings, including inpatient (ICU/non-ICU), outpatient, and individuals not seeking treatment
	Use patient flowchart similar to STROBE diagram^58^ reporting the number of patients eligible, excluded, and lost to follow-up (with reasons)
	Include a comparable cohort of individuals without COVID-19 for comparison
Recruitment strategy	Recruit patients consecutively and indicate reasons for any nonconsecutive enrollees
Follow-up	Define time zero, with universal reporting of time from initial diagnosis or first symptom onset
	Report mean length of follow-up, including SD and range
	Measure and report outcomes longitudinally at fixed intervals (at least monthly)
Exposure measurement	Report COVID-19 diagnosis based on PCR test or test of equivalent specificity
	Provide name of specific confirmatory test, along with its sensitivity and specificity
Outcomes of interest	Refer to established core outcome sets (eg, COMET Initiative^59^ or WHO COVID-19 Working Group^60^) to identify relevant symptoms and outcome definitions for a disease category
Outcome measurement	Report methods of collecting outcome information (eg, phone vs in-person); passive methods (eg, EHR) discouraged for symptoms unlikely to require specific treatment or be passively reported (eg, fatigue and neurocognitive outcomes)
	Include operational definitions for each measured symptom
	Report severity of symptoms (mild, moderate, severe, and/or critical)
	Report number of symptoms experienced by each patient
	Use validated instruments to measure symptoms when available (eg, Chalder Fatigue Scale or Fatigue Severity Scale to measure fatigue, 36-Item Short Form Survey or EuroQol questionnaires to measure quality of life) (Table 1)
	Include questionnaire used to measure symptoms (when applicable) in supplementary material
Results	Stratify symptom frequency and severity by baseline severity of COVID-19 infection and/or patient care setting and patient characteristics (eg, age, comorbidities, and race/ethnicity)

Abbreviations: CDC, Centers for Disease Control and Prevention; COMET, Core Outcome Measures in Effectiveness Trials; COPD, chronic obstructive pulmonary disease; EHR, electronic health record; ICU, intensive care unit; PCR, polymerase chain reaction; WHO, World Health Organization.

### Frequency of Persistent Outcomes

#### Symptom Persistence

Sixteen studies, most of which comprised patients who were previously hospitalized, reported the persistence of at least 1 symptom among their study population at last follow-up.^9,10,11,15,20,22,28,31,36,38,40,42,43,44,50,51^ This finding was common, with a median frequency of 72.5% (IQR, 55.0%-80.0%), and consistent, even among studies that followed up patients for almost 6 months (eg, 76% of patients in the Huang et al^22^ study and 84% of patients in the Taboada et al^43^ study) (eTable 6 in the Supplement).

#### Shortness of Breath or Dyspnea

The most frequently examined symptom was shortness of breath or dyspnea, with 26 studies reporting this outcome.^9,10,11,15,16,17,18,20,21,22,23,25,28,31,35,36,37,38,39,40,42,43,44,49,50,52^ Dyspnea was measured by self-reported data in 14 studies,^9,10,16,21,23,28,31,35,36,38,43,44,50,52^ by validated instruments (eg, the Patient-Reported Outcomes Measurement Information System Dyspnea Functional Limitations instrument^61^ or the modified Medical Research Council Dyspnea Scale^62^) in 10 studies,^15,17,20,22,25,37,39,40,42,49^ or by a combination of self-reported data and validated instruments in 2 studies.^11,18^ The median frequency of dyspnea was 36.0% (IQR, 27.6%-50.0%). Weerahandi et al^49^ reported the highest dyspnea frequency at 74.3%; however, 30.9% of the study population reported experiencing dyspnea before COVID-19 infection, although that subgroup reported substantial worsening of their baseline symptoms.^49^ Carvalho-Schneider et al^11^ and Garrigues et al^18^ reported dyspnea frequencies of 30.0% and 41.7%, respectively, based on self-report and frequencies of 7.7% and 29.0% based on a modified Medical Research Council Dyspnea Scale score of 2 or higher. This illustrates that frequencies can be substantially affected by changing outcome definitions even within the same study.

#### Fatigue or Exhaustion

Fatigue or exhaustion was examined by 25 studies^9,10,11,15,16,18,20,21,22,23,26,27,28,31,35,36,37,38,42,43,44,45,48,51,52^ and was frequently experienced by participants (median frequency, 40.0%; IQR, 31.0%-57.0%). Zhao et al^52^ reported a low frequency of 16.4%, but fatigue was determined retroactively using patients’ medical records. Three studies^23,37,45^ measured fatigue using validated instruments. Raman et al^37^ reported a fatigue frequency of 55% using the Fatigue Severity Scale^63^ (with a cutoff of ≥4 points), which is a 9-item questionnaire measuring the extent to which fatigue interferes with daily activities. Townsend et al^45^ found a frequency of 52.3% using the 11-item Chalder Fatigue Scale^64^ (with a cutoff of ≥4 points). Jacobs et al^23^ reported a frequency of 44.8% using the 10-item Patient-Reported Outcomes Measurement Information System Global Health instrument, which measures the severity of fatigue (none, mild or moderate, severe, and very severe).^65^ The remaining 22 studies^9,10,11,15,16,18,20,21,22,26,27,28,31,35,36,38,42,43,44,48,51,52^ did not specify how fatigue was defined; the median frequency of fatigue in these studies was 39.8% (IQR, 31.4%-59.0%).

#### Cough, Atypical Chest Pain, and Fever

Persistent cough was reported by 19 studies.^9,14,15,16,18,20,21,23,26,28,31,36,38,39,40,42,43,50,51^ Liang et al^26^ reported a frequency of 60%, but the remaining 18 studies reported a median frequency of 16.9% (IQR, 14.4%-25.1%). It is unclear why the findings from Liang et al^26^ were substantially different. Atypical chest pain was reported by 11 studies,^9,10,11,16,17,18,22,35,42,43,51^ and the reported frequencies were relatively consistent (median, 13.1%; IQR, 10.8%-18.0%). Fever was examined by 10 studies.^9,11,16,20,23,26,31,40,42,44^ Reported frequencies were relatively consistent across studies (median frequency, 1.0%; IQR: 0% to 3.0%).

#### Anosmia and Ageusia or Dysgeusia

Anosmia (loss of smell) was reported by 19 studies,^9,13,16,18,19,20,22,23,24,27,33,34,38,40,42,43,44,46,47^ and ageusia or dysgeusia (loss or distortion of taste) was reported by 13 studies.^16,18,19,20,22,23,27,33,34,38,42,44,47^ The reported persistence in some studies reflected the overall proportion of patients who experienced these symptoms persistently rather than the proportion of those who experienced symptoms that did not resolve after developing during the acute phase of infection. Seven studies^9,18,19,20,22,42,43^ did not report the number of patients experiencing the symptom at diagnosis. For the remaining studies, we recalculated frequencies to examine the probability of symptoms persisting if they had appeared during acute illness, as no study reported new loss of smell or taste after recovery. The median adjusted frequency was 23.6% (IQR, 12.4%-40.7%) for anosmia if this symptom occurred during the acute phase and 15.6% (IQR, 10.1%-23.9%) for persistent ageusia or dysgeusia. Including all studies, without adjustment, the corresponding median numbers for anosmia were 11% (IQR, 5.7%- 14.3%) and for ageusia or dysgeusia, 9% (IQR, 3.0%-11.2%).

#### Depression and/or Anxiety

Anxiety and/or depression was reported by 16 studies^8,15,17,20,21,22,27,28,29,30,37,42,44,48,50,51^; of those, 10 studies^15,17,20,28,29,30,37,44,48,51^ reported depression (median frequency, 14.9%; IQR, 11.0%-18.0%), and 10 studies^15,17,20,29,30,37,42,44,48,51^ reported anxiety (median frequency, 22.1%; IQR, 10.0%-29.6%). The frequencies of depression and anxiety were relatively consistent among studies that used standardized scales to measure those outcomes (Table 1). Xiong et al^51^ reported the lowest frequency of depression (4.3%); however, this study did not use a questionnaire or psychometric scale, and queries were limited to individuals who were willing and able to describe their symptoms. Three studies (Huang et al,^22^ Akter et al,^8^ and Halpin et al^21^) reported a combined prevalence of anxiety and depression of 21.1%, 21.6%, and 23.0%, respectively.

#### Cognitive Functioning

Cognitive outcomes were reported by 13 studies.^8,15,16,17,18,21,23,27,29,30,42,44,48^ Reported frequencies were relatively consistent across studies; 6 studies^15,16,17,42,44,48^ reported cognitive deficits (median frequency, 17.6%; IQR, 15.0%-21.6%), 5 studies^8,18,21,27,42^ reported loss of memory (median frequency, 28.3%; IQR, 18.6%-35.8%), and 4 studies^8,18,21,42^ reported difficulty concentrating (frequency, 22.0%, 25.4%, 25.6%, and 28.0%).

#### Composite Quality of Life

Four studies^9,12,20,49^ reported physical and mental health composite scores. Arnold et al^9^ and Chen et al^12^ measured these outcomes using the 36-Item Short Form Survey, in which a score of 100 represents the best possible health status. These 2 studies reported comparable composite scores, with mean scores of 40.2 and 55.9 for physical health and 44.8 and 48.9 for mental health, respectively. Weerahandi et al^49^ used the PROMIS Global Health-10 instrument^61^ and Gonzalez et al^20^ used the 12-Item Short Form survey, converting raw scores to normalized *t* scores; these scores are standardized such that a mean (SD) score of 50 (10) points represents the general US population. Weerahandi et al^49^ reported a mean (SD) of 43.8 (9.3) points for physical health and 47.3 (9.3) points for mental health, and Gonzalez et al^20^ reported a median of 45.9 points (IQR, 36.1-54.4 points) for physical health and 55.5 points (IQR, 40.6-58.0 points) for mental health; these scores were comparable to those reported by Arnold et al^9^ and Chen et al.^12^

## Discussion

This systematic review found that persistent COVID-19 symptoms were common, with 72.5% of patients reporting at least 1 symptom at 60 days or more after diagnosis, symptom onset, or hospitalization or at 30 days or more after recovery from acute illness or hospital discharge. This finding was consistent even among studies that followed up patients for almost 6 months,^22,43^ suggesting that symptoms may persist long after recovery among some patients. Most patients reported thus far were previously hospitalized. This finding suggests that inclusion of the prolonged burden of morbidity is warranted for future research on the overall health implications of the pandemic.

The most frequently reported persistent symptoms were fatigue and shortness of breath, both of which can be debilitating. Atypical chest pain was reported in approximately 1 of 7 patients. Inability to concentrate, informally described as brain fog, was only examined in 4 studies^8,18,21,42^ and was experienced by approximately 1 in 4 patients. Other neurocognitive deficits had similar frequencies. These observations are consistent with imaging and pathophysiologic measurements indicating persistent COVID-19 structural and functional organ system abnormalities. Three studies included in this review combined symptom measurements with magnetic resonance imaging scans of various organs. Raman et al^37^ reported tissue abnormalities in the lungs (60%), kidneys (29%), heart (26%), and liver (10%). Lu et al^27^ found that patients with COVID-19 were more likely to have brain abnormalities, including abnormalities in regions associated with loss of smell and memory, compared with healthy individuals. Puntmann et al^35^ reported that 78% of patients with COVID-19 had heart abnormalities, suggesting frequent myocardial inflammation.

Although most studies did not stratify outcomes by age, 30 of the 45 studies with age information reported mean or median ages younger than 60 years; in 14 studies, mean or median ages were 50 years or younger. This finding suggests that, among cases requiring hospitalization, younger age did not protect against prolonged symptoms.

### Limitations

This study has limitations. Design limitations among the included studies prevented us from addressing several important issues, including the duration of persistent symptoms, the percentage of symptoms that were ultimately resolved, and the long-term trajectory of global quality of life and function. We had limited data on the persistence of symptoms by initial severity, particularly among outpatients. Because many symptoms were not captured using standardized definitions or instruments, it was difficult to compare frequency and severity. Studies that measured the same symptom in different ways reported substantially different estimates, even within the same study. Few of the studies examined past history or baseline prevalence of similar symptoms or assessed prevalence in a contemporaneous group that did not have COVID-19, making it difficult to assess the fraction or severity of persistent symptoms that could be associated with COVID-19 infection.

Many features associated with combinability of estimates are not markers of study quality. For example, if the definition of time zero varies substantially among studies, particularly in combination with other time dimensions, then the final estimates cannot be combined to increase precision. The only feature that was unequivocally a measure of quality rather than design was the extent of patient retention, which exceeded 80% in only 15 of 45 studies (33.3%), indicating that quality was no better than moderate (ie, retention was >80%) based on this measure alone.

This heterogeneity of design features and quality emphasizes the importance of improving and standardizing methods used in future studies. We provide recommendations in Table 3 to improve information quality and design consistency, thereby increasing the comparability and validity of results with regard to study population, recruitment strategy, follow-up, exposure measurement, outcomes of interest, and outcome measurement.

## Conclusions

This systematic review found that COVID-19 symptoms frequently persist beyond the acute phase of infection, but there is a need to standardize designs and improve study quality. With millions of individuals experiencing COVID-19 infection, persistent symptoms are a burden on individual patients and their families as well as on outpatient care, public health, and the economy. The designs of studies reported to date preclude making precise risk estimates about many long-term outcomes, particularly by patient or disease characteristic, but they suggest that the problem of persistent symptoms is substantial. The findings of this review should help to improve future study quality and reduce heterogeneity in study design and reporting, enabling researchers to better assess the risk of long-term outcomes associated with COVID-19 and physicians to better advise and treat their patients.

## Notes

Supplement. **eTable 1.** Literature Search Strategy **eTable 2.** Studies Excluded From Review **eTable 3.** Study and Patient Characteristics **eTable 4.** Selection Criteria **eTable 5.** Follow-Up and Outcome Measurement **eTable 6.** Reported Outcomes and Frequencies at Follow-Up **eFigure.** MOOSE Flowchart for the Literature Search eReferences
