# Supplementary material for: Assessment of the Frequency and Variety of Persistent Symptoms Among Patients With COVID-19: A Systematic Review
Source: JAMA Netw Open. 2021 May 26;4(5):e2111417. doi: 10.1001/jamanetworkopen.2021.11417 (PMC8155823; doi:10.1001/jamanetworkopen.2021.11417)
Supplement: Supplement. — eTable 1. Literature Search Strategy eTable 2. Studies Excluded From Review eTable 3. Study and Patient Characteristics eTable 4. Selection Criteria eTable 5. Follow-Up and Outcome Measurement eTable 6. Reported Outcomes and Frequencies at Follow-Up eFigure. MOOSE Flowchart for the Literature Search eReferences [file jamanetwopen-e2111417-s001.pdf]

Supplementary Online Content

Nasserie T, Hittle M, Goodman SN. Assessment of the frequency and variety of persistent symptoms among patients with COVID-19: a systematic review. *JAMA Netw Open*. 2021;4(5):e2111417. doi:10.1001/jamanetworkopen.2021.11417

eTable 1. Literature Search Strategy

eTable 2. Studies Excluded From Review

eTable 3. Study and Patient Characteristics

eTable 4. Selection Criteria

eTable 5. Follow-Up and Outcome Measurement

eTable 6. Reported Outcomes and Frequencies at Follow-Up

eFigure. MOOSE Flowchart for the Literature Search

eReferences

This supplementary material has been provided by the authors to give readers additional information about their work.

© 2021 Nasserie T et al. *JAMA Network Open*.

eTable 1. Literature Search Strategy

Database	Terms/Code
PubMed	(((COVID-19) OR (SARS-CoV-2) OR (coronavirus) OR (2019-nCoV)) AND ((long-term) OR ("long term") OR ("long haul*") OR ("after recovery") OR (prolong*) OR (persist*)) AND ((outcome*) OR (symptom*) OR (disease*) OR (illness*)) AND ((cohort) OR (follow up) OR (longitudinal)))
Web of Science	Query #1: TS=((COVID-19) OR (SARS-CoV-2) OR (coronavirus) OR (2019-nCoV))<br>Query #2: TS=((long-term) OR ("long term") OR ("long haul*") OR ("after recovery") OR (prolong*) OR (persist*))<br>Query #3: TS=((outcome*) OR (symptom*) OR (disease*) OR (illness*))<br>Query #4: TS=((cohort) OR (follow up) OR (longitudinal))<br>Final query: #1 AND #2 AND #3 AND #4

© 2021 Nasserie T et al. *JAMA Network Open*.

eTable 2. Studies Excluded from Review

**Reference**	**Summary Comment for Exclusion**
Shang Y, Xu C, Jiang F, et al. Clinical characteristics and changes of chest CT features in 307 patients with common COVID-19 pneumonia infected SARS-CoV-2: A multicenter study in Jiangsu, China. *Int J Infect Dis IJID Off Publ Int Soc Infect Dis*. 2020;96:157-162. doi:10.1016/j.ijid.2020.05.006	Insufficient follow-up length (maximum of to 3 weeks after symptom onset)
Boscolo-Rizzo P, Borsetto D, Fabbris C, et al. Evolution of altered sense of smell or taste in patients with mildly symptomatic COVID-19. JAMA Otolaryngol-- Head Neck Surg. 2020;146(8):729-732. doi:10.1001/jamaoto.2020.1379	Insufficient follow-up length (4 weeks after symptom onset)
Guo T, Fan Y, Chen M, et al. Cardiac complications in patients hospitalised with COVID-19. *JAMA Cardiol.* 2020;5(7):811-818. doi: 10.1001/jamacardio.2020.1017	Does not assess prevalence of persistent symptoms
Cellai M, O'Keefe JB. Characterization of Prolonged COVID-19 Symptoms in an Outpatient Telemedicine Clinic. Open Forum Infect Dis. 2020;7(10):ofaa420. doi:10.1093/ofid/ofaa420	Insufficient follow-up length (6 weeks after symptom onset)
Chung TW-H, Sridhar S, Zhang AJ, et al. Olfactory Dysfunction in Coronavirus Disease 2019 Patients: Observational Cohort Study and Systematic Review. *Open Forum Infect Dis*. 2020;7(6):ofaa199. doi:10.1093/ofid/ofaa199	Insufficient follow-up length (7-9 days after symptoms subsided)
Cicco S, Vacca A, Cittadini A, Marra AM. Long-Term Follow-Up May be Useful in Coronavirus Disease 2019 Survivors to Prevent Chronic Complications. Infect Chemother. 2020;52(3):407-409. doi:10.3947/ic.2020.52.3.407	Review of current knowledge about chronic complications among survivors
Frija-Masson J, Debrary MP, Gilbert M, et al. Functional characteristics of patients with SARS-CoV-2 pneumonia at 30 days post-infection. *Eur Respir J*. 2020;56(2). doi: 10.1183/13993003.01754-2020	Insufficient follow-up length (30 days after infection)

© 2021 Nasserie T et al. *JAMA Network Open*.

George PM, Barratt SL, Condliffe R, et al. Respiratory follow-up of patients with COVID-19 pneumonia. *Thorax*. 2020, doi: 10.1136/thoraxjnl-2020-215314	Review of current knowledge about long-term respiratory outcomes
Karimi-Galougahi M, Safavi Naini A, Ghorbani J, Raad N, Raygani N. Emergence and Evolution of Olfactory and Gustatory Symptoms in Patients with COVID-19 in the Outpatient Setting. *Indian J Otolaryngol Head Neck Surg*. Published online September 28, 2020:1-7. doi:10.1007/s12070-020-02166-4	Insufficient follow-up length (two weeks after initial symptoms)
Khoo B, Tan T, Clarke SA, et al. Thyroid function before, during and after COVID-19. *J Clin Endocrinol Metab*. Published online November 12, 2020. doi:10.1210/clinem/dgaa830	Insufficient length of follow-up for some patients (IQR is 52 to 108 days after hospital admission)
Konstantinidis I, Delides A, Tsakiropoulou E, Maragoudakis P, Sapounas S, Tsiodras S. Short-term follow-up of self-isolated COVID-19 patients with smell and taste dysfunction in Greece: Two phenotypes of recovery. ORL. Published online October 13, 2020:1-9. doi:10.1159/000511436	Insufficient follow-up length (4 weeks after diagnosis)
Mizrahi B, Shilo S, Rossman H, et al. Longitudinal symptom dynamics of COVID-19 infection. *Nature Comm*. 2020:11, 6208. doi: 10.1038/s41467-020-20053-y	Insufficient follow-up length (mean 31.4 ± 20.1 days after discharge)
Małek ŁA, Marczak M, Miłosz-Wieczorek B, et al. Cardiac involvement in consecutive elite athletes recovered from Covid-19: A magnetic resonance study. *J Magn Reson Imaging JMRI*. Published online January 20, 2021. doi:10.1002/jmri.27513	Insufficient follow-up length (median: 32 days after diagnosis, IQR: 22 to 62 days)
Manson JJ, Crooks C, Naja M, et al. COVID-19-associated hyperinflammation and escalation of patient care: a retrospective longitudinal cohort study. *Lancet Rheumatol*. 2020;2(10):e594-e602. doi:10.1016/S2665-9913(20)30275-7	Length of follow-up is not clearly reported. Minimum follow time is provided, but not range

Mazza MG, De Lorenzo R, Conte C, et al. Anxiety and depression in COVID-19 survivors: Role of inflammatory and clinical predictors. *Brain Behav Immun*. 2020;89:594-600. doi:10.1016/j.bbi.2020.07.037	Insufficient follow-up length (mean 31.29 +/ 15.7 days after discharge)
Negrini F, Ferrario I, Mazziotti D, et al. Neuropsychological features of severe hospitalized COVID-19 patients at clinical stability and clues for post-acute rehabilitation. Arch Phys Med Rehabil. Published online September 26, 2020. doi:10.1016/j.apmr.2020.09.376	Insufficient follow-up length (minimum of 30 days after hospital admission)
Paderno A, Mattavelli D, Rampinelli V, et al. Olfactory and gustatory outcomes in COVID-19: A prospective evaluation in nonhospitalized subjects. Otolaryngol Head Neck Surg. Published online June 30, 2020. doi:10.1177/0194599820939538	Insufficient follow-up length (maximum 45 days after symptom onset)
Panda S, Mohamed A, Sikka K, et al. Otolaryngologic Manifestation and Long-Term Outcome in Mild COVID-19: Experience from a Tertiary Care Centre in India. *Indian J Otolaryngol Head Neck Surg Off Publ Assoc Otolaryngol India*. Published online October 14, 2020:1-6. doi:10.1007/s12070-020-02217-w	Insufficient follow-up length (1 month after hospital admission)
Pellaud C, Grandmaison G, Pham Huu Thien HP, et al. Characteristics, comorbidities, 30-day outcome and in-hospital mortality of patients hospitalised with COVID-19 in a Swiss area - a retrospective cohort study. *Swiss Med Wkly*. 2020;150:w20314. doi:10.4414/smw.2020.20314	Insufficient follow-up length (7-9 days after symptom onset)
Prescott C, Sussman JB, Joost Wiersinga W. Post-critical illness vulnerability. Curr Opin Crit Care. 2020;26(5):500-507. doi: 10.1097/MCC.0000000000000076	Review of current knowledge about long-term outcomes among critical illness survivors
Rajpal S, Tong MS, Borchers J, et al. Cardiovascular Magnetic Resonance Findings in Competitive Athletes Recovering From COVID-19 Infection. JAMA Cardiol. Published online September 11, 2020. doi:10.1001/jamacardio.2020.4916	Insufficient follow-up length (some patients only followed for 11 days after diagnosis)

© 2021 Nasserie T et al. *JAMA Network Open*.

Shaw B, Daskareh M, Gholamrezanezhad A. The lingering manifestations of COVID-19 during and after convalescence: update on long-term pulmonary consequences of coronavirus disease 2019 (COVID-19). Radiol Med (Torino). Published online October 1, 2020. doi:10.1007/s11547-020-01295-8	Review of current knowledge about long-term pulmonary outcomes
Tenforde MW. Symptom Duration and Risk Factors for Delayed Return to Usual Health Among Outpatients with COVID-19 in a Multistate Health Care Systems Network — United States, March–June 2020. *MMWR Morb Mortal Wkly Rep*. 2020;69. doi:10.15585/mmwr.mm6930e1	Insufficient follow-up length (14-21 days after test date)
Wang X, Xu H, Jiang H, et al. Clinical features and outcomes of discharged coronavirus disease 2019 patients: a prospective cohort study. *QJM Mon J Assoc Physicians*. 2020;113(9):657-665. doi:10.1093/qjmed/hcaa178	Insufficient follow-up length (some patients only followed for 3 weeks)
Xia L, Chen J, Friedemann T, et al. The Course of Mild and Moderate COVID-19 Infections-The Unexpected Long-Lasting Challenge. *Open Forum Infect Dis*. 2020;7(9):ofaa286. doi:10.1093/ofid/ofaa286	Insufficient follow-up length (minimum of one month after hospital admission)
Yan CH, Prajapati DP, Ritter ML, DeConde AS. Persistent Smell Loss Following Undetectable SARS-CoV-2. *Otolaryngol Head Neck Surg*. Published online June 9, 2020:194599820934769. doi:10.1177/0194599820934769	Insufficient follow-up length (average of 16.2 days after diagnosis, IQR (9-22.3 days)
Li Y, Li M, Wang M, et al. Acute cerebrovascular disease following COVID-19: a single center, retrospective, observational study. Stroke Vasc Neurol. 2020;5(3):279-284. doi:10.1136/svn-2020-000431	Length of follow-up is not clearly reported
Yang SS, Lipes J, Dial S, et al. Outcomes and clinical practice in patients with COVID-19 admitted to the intensive care unit in Montréal, Canada: a descriptive analysis. *CMAJ Open*. 2020;8(4):E788-E795. doi:10.9778/cmajo.20200159	Does not assess prevalence of persistent symptoms

© 2021 Nasserie T et al. *JAMA Network Open*.

Zou R, Chen F, Chen D, Xu C-L, Xiong F. Clinical characteristics and outcome of hemodialysis patients with COVID-19: a large cohort study in a single Chinese center. Ren Fail. 2020;42(1):950-957. doi:10.1080/0886022X.2020.1816179	Insufficient follow-up length (3 weeks for some patients)
Hopkins C, Surda P, Vaira LA, et al. Six month follow-up of self-reported loss of smell during the COVID-19 pandemic. Rhinology. Published online December 15, 2020. doi:10.4193/Rhin20.544	Study sample was not limited to individuals who tested positive for COVID-19
Goërtz YMJ, Van Herck M, Delbressine JM, et al. Persistent symptoms 3 months after a SARS-CoV-2 infection: the post-COVID-19 syndrome? ERJ Open Res. 2020;6(4). doi:10.1183/23120541.00542-2020	Selected for patients with existing persistent symptoms
Lovato A, Galletti C, Galletti B, de Filippis C. Clinical characteristics associated with persistent olfactory and taste alterations in COVID-19: A preliminary report on 121 patients. Am J Otolaryngol. 2020;41(5):102548. doi:10.1016/j.amjoto.2020.102548	Insufficient follow-up length (mean of 38 days after diagnosis)
Sheng W-H, Liu W-D, Wang J-T, Chang S-Y, Chang S-C. Dysosmia and dysgeusia in patients with COVID-19 in northern Taiwan. J Formos Med Assoc Taiwan Yi Zhi. 2021;120(1 Pt 2):311-317. doi:10.1016/j.jfma.2020.10.003	Assesses median duration of symptoms, not prevalence of persistent symptoms
Reiter ER, Coelho DH, Kons ZA, Costanzo RM. Subjective smell and taste changes during the COVID-19 pandemic: Short term recovery. Am J Otolaryngol. 2020;41(6):102639. doi:10.1016/j.amjoto.2020.102639	Insufficient follow-up length (1 month after symptom onset)
Han X, Fan Y, Alwalid O, et al. Six-Month Follow-up Chest CT findings after Severe COVID-19. Pneumonia. Radiology. Published online January 26, 2021:203153. doi:10.1148/radiol.2021203153	Assessing outcomes among individuals with pneumonia

© 2021 Nasserie T et al. *JAMA Network Open*.

Brandão Neto D, Fornazieri MA, Dib C, et al. Chemosensory Dysfunction in COVID-19: Prevalences, Recovery Rates, and Clinical Associations on a Large Brazilian Sample. Otolaryngol--Head Neck Surg Off J Am Acad Otolaryngol-Head Neck Surg. Published online September 1, 2020:194599820954825. doi:10.1177/0194599820954825	Insufficient follow-up length for some patients (36 to 119 days after symptom onset)
Liu D, Baumeister RF, Veilleux JC, et al. Risk factors associated with mental illness in hospital discharged patients infected with COVID-19 in Wuhan, China. Psychiatry Res. 2020;292:113297. doi:10.1016/j.psychres.2020.113297	Length of follow-up unclear (minimum not reported)
Udwadia ZF, Koul PA, Richeldi L. Post-COVID lung fibrosis: The tsunami that will follow the earthquake. *Lung India*. 2021;38(Supplement):S41-S47. doi:10.4103/lungindia.lungindia_818_20	Review of current knowledge about long term pulmonary outcomes
Trinkmann F, Müller M, Reif A, et al. Residual symptoms and lower lung function in patients recovering from SARS-CoV-2 infection. *Eur Respir J*. 2021;57(2). doi:10.1183/13993003.03002-2020	Insufficient follow up length (68 ± 16 days after symptom onset)
Makaronidis J, Firman C, Magee CG, et al. Distorted chemosensory perception and female sex associate with persistent smell and/or taste loss in people with SARS-CoV-2 antibodies: a community based cohort study investigating clinical course and resolution of acute smell and/or taste loss in people with and without SARS-CoV-2 antibodies in London, UK. *BMC Infect Dis*. 2021;21(1):221. doi:10.1186/s12879-021-05927-w	Insufficient follow up length (4-6 weeks after positive test)

© 2021 Nasserie T et al. *JAMA Network Open*.

Lampl BMJ, Buczovsky M, Martin G, Schmied H, Leitzmann M, Salzberger B. Clinical and epidemiological data of COVID-19 from Regensburg, Germany: a retrospective analysis of 1084 consecutive cases. *Infection*. Published online March 5, 2021. doi:10.1007/s15010-021-01580-2	Insufficient follow up length (6 weeks after symptom onset)
Jaywant A, Vanderlind WM, Alexopoulos GS, Fridman CB, Perlis RH, Gunning FM. Frequency and profile of objective cognitive deficits in hospitalized patients recovering from COVID-19. *Neuropsychopharmacology*. Published online February 15, 2021. doi:10.1038/s41386-021-00978-8	Insufficient follow up length (43 days after hospital admission)
Zhou M, Wong C-K, Un K-C, et al. Cardiovascular sequelae in uncomplicated COVID-19 survivors. *PLoS One*. 2021;16(2):e0246732. doi:10.1371/journal.pone.0246732	Insufficient follow up length (1-4 weeks after hospital discharge)
Logue JK, Franko NM, McCulloch DJ, et al. Sequelae in Adults at 6 Months After COVID-19 Infection. *JAMA Netw Open*. 2021;4(2):e210830. doi:10.1001/jamanetworkopen.2021.0830	Insufficient follow up length (less than 30 days or 30-60 days after symptom onset for some patients)
Hall J, Myall K, Lam JL, et al. Identifying patients at risk of post-discharge complications related to COVID-19 infection. *Thorax*. Published online February 4, 2021. doi:10.1136/thoraxjnl-2020-215861	Selected for patients with persistent symptoms
Stavem K, Ghanima W, Olsen MK, Gilboe HM, Einvik G. Persistent symptoms 1.5-6 months after COVID-19 in non-hospitalised subjects: a population-based cohort study. *Thorax*. Published online December 3, 2020. doi:10.1136/thoraxjnl-2020-216377	Insufficient follow up length (1.5-6 months after symptom onset)

© 2021 Nasserie T et al. *JAMA Network Open*.

Leite VF, Rampim DB, Jorge VC, et al. Persistent symptoms and disability after COVID-19 hospitalization: data from a comprehensive telerehabilitation program. *Arch Phys Med Rehabil*. Published online March 9, 2021. doi:10.1016/j.apmr.2021.03.001	Insufficient follow up length (21.8±11.7 days after hospital discharge)

© 2021 Nasserie T et al. *JAMA Network Open*.

eTable 3. Study and Patient Characteristics

Study	Country	Cohort Type, Study Arms	Diagnostic Criteria	Severity of COVID-19 illness				Patient Demographics & Underlying Comorbidities		
				Asymp.- %	*Mild or Moderate - %*	*Severe - %*	*Critical - %*	*Age in Years - Mean (SD)*	*Male - %*	**BMI & Comorbidities**
Akter[1]	Bangladesh	Non-concurrent, single-arm	Positive SARS-CoV-2 real-time reverse transcriptase PCR	Not reported				NR	76.0	Diabetes: 19.89%<br>Cancer: 1.4%<br>Cardiovascular diseases: 9.1%<br>Respiratory disease: 6.1%<br>Liver diseases: 2.2%<br>Heart attack history: 2.5%<br>Other chronic diseases: 7.9%
Arnold[2]	United Kingdom	Concurrent, single-arm	Positive SARS-CoV-2 real-time reverse transcriptase PCR or radiological diagnosis	0	84	16	0	47 (range: 32 to 61)	61.8	Hypertension: 15%<br>Mean BMI (kg/m^2): 31.2<br>Heart disease: 22%<br>Chronic lung disease: 15%<br>Severe liver disease: 0%<br>Severe kidney disease: 3.7%<br>HIV/AIDS: 0%
Carfi[3]	Italy	Concurrent, single-arm	Positive SARS-CoV-2 real-time reverse transcriptase PCR	Not reported				56.5 (14.6)	62.9	Diabetes: 7%<br>Hypertension: 35%<br>BMI (kg/m^2), mean SD: 26.3 (4.4)<br>Chronic heart disease: 4.9%<br>Atrial fibrillation: 2.8%<br>Heart failure: 2.8%<br>Stroke: 1.4%<br>Kidney failure: 2.1%<br>Thyroid disease: 18.2%<br>COPD: 9.1%<br>Active cancer: 3.5%<br>Immune disorders: 11.2%

© 2021 Nasserie T et al. *JAMA Network Open*.

Carvalho-Schneider[4]	France	Concurrent, single-arm	Positive SARS-CoV-2 real-time reverse transcriptase PCR	0	78	22	0	49 (15)	47.7	Comorbid conditions:<br>None: 49.1%<br>1: 31.5%<br>2 or more: 19.2%
Chen[5]	China	Concurrent, single-arm	Lab-confirmed	0	91	9	0	47.2 (13.0)	51.5	BMI, kg/m^2, mean (SD): 23.64 (3.31)<br>History of chronic disease: 31.9%
Chiesa-Estomba[6]	Spain	Concurrent, single-arm	Positive SARS-CoV-2 real-time reverse transcriptase PCR or positive immunoglobulin G/M test	0	100	0	0	41 (13)	36.5	Diabetes: 2.5%<br>Heart problems: 2.3%<br>Chronic obstructive pulmonary disorder: 0.8%<br>Hypertension: 6.3%<br>Asthma: 5.6%<br>Hypothyroidism: 6.1%<br>Autoimmune disease: 3.9%
Chopra[7]	United States	Concurrent, single-arm	Not stated	Not reported				Median: 62 (IQR: 50-72)	51.8	Diabetes: 34.9%<br>Hypertension: 64%<br>Cardiovascular disease: 24.1%<br>Moderate/severe kidney disease: 23%<br>Asthma: 13.4%<br>Congestive heart failure/cardiomyopathy: 11.6%<br>Chronic obstructive pulmonary disease: 10.4%<br>Cerebrovascular disease/paraplegia: 10.4%<br>Dementia: 7.7%<br>Cancer: 7.1%<br>None: 14.3%

© 2021 Nasserie T et al. *JAMA Network Open*.

D'Cruz[8]	United Kingdom	Concurrent, single-arm	Positive SARS-CoV-2 real-time reverse-transcriptase PCR	0	0	66	34	58.7 (14.4)	62.2	Median BMI ((kg/m^2): 30.0 (IQR: 25.9 to 35.2)<br>Current or former smoker: 25.4%<br>Cardiovascular disease: 45.4%<br>Diabetes: 34.5%<br>Immunosuppressed: 13.4%<br>Obstructive lung disease: 10.9%<br>Malignancy: 10.1%<br>End-stage renal failure: 6.7%<br>Thyroid disease: 5.9%<br>Mental health conditions: 5.0%<br>Cerebrovascular disease: 4.2%
Daher[9]	Germany	Concurrent, single-arm	Positive SARS-CoV-2 real-time reverse transcriptase PCR	Not reported				64 (3)	66.7	Hypertension: 59%<br>Diabetes mellitus: 25%<br>COPD: 9%<br>Bronchial asthma: 13%<br>Heart failure: 9%<br>Atrial fibrillation: 9%<br>Chronic kidney disease: 22%<br>Coronary artery disease: 19%
de Graaf[10]	Netherlands	Concurrent, single-arm	Positive SARS-CoV-2 real-time reverse-transcriptase PCR	Not reported				60.8 (13)	63.0	BMI: 27.8 ± 4.5<br>Hypertension: 34%<br>Hypercholesterolemia: 27%<br>Diabetes: 23%<br>Smoking: 11%<br>Alcohol: 23%<br>Cardiovascular disease: 28%<br>Heart failure: 1%<br>Atrial fibrillation: 5%<br>Valvular heart disease: 6%<br>Myocardial infarction: 4%<br>Stroke or transient ischemic attack: 10%<br>Peripheral vascular disease:

© 2021 Nasserie T et al. *JAMA Network Open*.

										2%<br>Chronic kidney injury: 11%
Garrigues[11]	France	Concurrent, single-arm	Positive SARS-CoV-2 real-time reverse transcriptase PCR and/or abnormalities on chest CT scan	Not reported				63.2 (15.7)	62.5	Hypertension: 46.7%<br>Diabetes: 21.7%<br>BMI (kg/m^2):<br><25: 29.2%<br>>/ 25: 47.5%<br>Missing: 23.3%
Gherlone[12]	Italy	Concurrent, single-arm	Positive SARS-CoV-2 real-time reverse-transcriptase PCR	0	0	75.4	24.6	Median: 62.5 (IQR: 53.9 to 74.1)	75.4	Hypertension: 41%<br>Coronary artery disease: 9.8%<br>Diabetes mellitus: 13.9%<br>Chronic kidney disease: 7.4%<br>Neoplasia: 5.7%<br>Chronic obstructive pulmonary disease: 6.6%<br>Smoking: 39.3%
Gonzalez[13]	Spain	Concurrent, single-arm	Not reported	0	0	0	100	Median: 60 (IQR: 48 to 65)	74.2	Median BMI ((kg/m^2): 28.2 (IQR: 25.4 to 32.6)<br>Current or former smoker: 56.7%<br>Hypertension: 37.1%<br>Diabetes mellitus: 14.5%<br>Chronic heart disease: 9.7%<br>Asthma: 4.8%<br>COPD: 4.8%
Halpin[14]	United Kingdom	Concurrent, single-arm	Positive SARS-CoV-2 real-time reverse transcriptase PCR	Not reported				Ward - Median: 70.5 (range: 20 to 93)<br>ICU - Median: 58.5 (range: 34 to 84)	54.0	BMI:<br>Underweight: 3%<br>Healthy weight: 25%<br>Overweight: 35%<br>Obese: 24%<br>Unknown: 13%

© 2021 Nasserie T et al. *JAMA Network Open*.

										Active cancer: 7%<br>Active or previous cancer: 21%<br>Heart failure: 5%<br>Hyperlipidemia: 4%<br>Ischemic heart disease: 10%<br>Hypertension: 41%<br>Tachyarrhythmias: 11%<br>Valvular heart disease: 3%<br>Venous thromboembolism:5%
Huang[15]	China	Concurrent, single-arm	Labconfirmed	0	25.3	70.7	4	Median: 57 (IQR: 47 to 65)	51.8	Hypertension: 29%<br>Diabetes: 12%<br>Cardiovascular diseases: 7%<br>Cerebrovascular diseases: 3%<br>Malignant tumour: 3%<br>COPD: 2%<br>Chronic kidney disease: 2%<br>Past smoker: 3%<br>Current smoker: 6%
Jacobs[16]	United States	Concurrent, single-arm	Positive SARS-CoV-2 real-time transcriptase PCR	0	87.4	12.6	0	Median: 57 (IQR: 48 to 68)	60.9	Overweight: 36.1%<br>Obese: 49.2%<br>Hypertension: 47.5%<br>Diabetes: 28.4%<br>CAD or history of MI: 11.5%<br>Arrhythmia: 4.9%<br>Heart failure: 2.7%<br>Hyperlipidemia: 10.9%<br>Asthma: 10.4%<br>Cancer: 9.8%<br>Immunodeficiency: 4.4%<br>Hypothyroidism: 4.4%<br>Psychiatric disorders: 4.4%<br>Obstructive sleep apnea: 3.3%<br>COPD: 3.8%<br>Thromboembolic disease: 1.6%

© 2021 Nasserie T et al. *JAMA Network Open*.

Lechien[17]	Belgium	Concurrent, single-arm	Positive SARS-CoV-2 real-time transcriptase PCR	0	100	0	0	46.2 (11.2)	33.0	Diabetes: 2.7%<br>Hypertension: 6.8%<br>Asthma: 6.8%<br>Reflux: 10.8%<br>Allergic rhinitis: 6.8%<br>Hypothyroidism: 2.7%
Lerum[18]	Norway	Concurrent, single-arm	Diagnostic codes U07.1, U07.2, or J12.x	Not reported				Median: 59 (IQR: 49 to 72)	52.4	Median BMI (IQR): 25.8 (23.9 to 29.6)<br>Current smoker: 3.4%<br>Past smoker: 39%<br>Hypertension: 35%<br>Diabetes: 8%
Liang[19]	China	Concurrent, single-arm	Positive SARS-CoV-2 real-time reverse transcriptase PCR	0	91	9		41.3 (13.8)	27.6	Hypertension: 6.6%<br>Cardiovascular disease: 1.2%<br>Diabetes: 3.9%<br>Thyroid disease: 3.6%<br>Pulmonary disease: 14.5%<br>Digestive system disease: 18.4%<br>Smoking: 0%
Lu[20]	China	Concurrent, two-arm	Positive SARS-CoV-2 real-time reverse transcriptase PCR	0	78	20	2	44.1 (16)	56.7	Hypertension: 21.67%<br>Diabetes: 10.00%
Mandal[21]	United Kingdom	Concurrent, single-arm	Positive SARS-CoV-2 real-time reverse transcriptase PCR	0	67	30	0	59.9 (16.1)	62.0	Hypertension: 41%<br>Diabetes mellitus: 27.2%<br>Asthma and/or COPD: 17.5%<br>Chronic kidney disease: 11%<br>Ischaemic heart disease: 9.7%<br>Obese: 8%<br>None: 34%
Mazza[22]	Italy	Concurrent, single-arm	Positive SARS-CoV-	Not reported	58.5 (12.8)	65.9	Not reported			

© 2021 Nasserie T et al. *JAMA Network Open*.

			2 real-time reverse-transcriptase PCR							
Mendez[23]	Spain	Non-concurrent, single-arm	Positive SARS-CoV-2 real-time reverse-transcriptase PCR	Not reported				Median: 57 (IQR: 49 to 67)	58.7	Hypertension: 32.4%<br>Diabetes: 16.2%<br>Dyslipidemia: 29/1%<br>Chronic heart disease: 5.6%<br>Chronic renal disease: 1.7%<br>Chronic liver disease: 1.7%<br>Cancer: 1.7%<br>Chronic respiratory disease: 11.7%
Moreno-Perez[24]	Spain	Concurrent, single-arm	Positive SARS-CoV-2 real-time reverse transcriptase PCR	0	34.3	65.7	0	Median: 62 (range: 53 to 72)	52.7	Hypertension: 36.5%<br>Diabetes: 11.6%<br>Obesity: 30.6%<br>Cardiovascular disease: 6.9%<br>Chronic respiratory disease: 18.1%<br>Immunosuppression: 4.1%
Munro[25]	United Kingdom	Concurrent, single-arm	Positive SARS-CoV-2 real-time reverse transcriptase PCR or chest radiology consistent with COVID-19	Not reported				Not reported	NR	Not reported
Nguyen[26]	France	Concurrent, single-arm	Positive SARS-CoV-2 real-time reverse transcriptas	Not reported				Median: 36 (IQR: 27 to 48)	44.8	Hypertension: 12.8%<br>Diabetes: 9.6%<br>Chronic respiratory disease: 12%<br>Chronic cardiac disease: 2.4%

© 2021 Nasserie T et al. *JAMA Network Open*.

			e PCR or serological test							Cancer: 0.8%<br>Obesity: 9.6%
Poncet-Megemont[27]	France	Non-concurrent, single-arm	Lab confirmed or based on chest CT scan	0	59	37	4	48.5 (15.3)	37.4	Not reported
Puntmann[28]	Germany	Concurrent, two-arm	Positive SARS-CoV-2 real-time reverse transcriptase PCR	18	49	33	0	49 (14)	53.0	Hypertension: 22%<br>Diabetes: 18%<br>BMI, (kg/m^2), median (IQR): 25 (23 to 28)<br>Hypercholesterolemia: 22%<br>Known coronary artery disease: 135<br>Smoking: 22%<br>COPD or asthma: 21%
Qu[29]	China	Concurrent, single-arm	Positive SARS-CoV-2 real-time reverse-transcriptase PCR	0	90.6	9.4	0	Median: 47.5 (IQR: 37 to 57)	50.0	Not reported
Raman[30]	United Kingdom	Concurrent, two-arm	Positive SARS-CoV-2 real-time reverse transcriptase PCR	Not reported				55.4 (13.2)	58.6	Hypertension: 37.9%<br>Diabetes: 15.5%<br>Current/ex smoker: 34.5%<br>Coronary artery disease: 3.4%<br>Cerebrovascular disease: 1.7%<br>Asthma: 34.5%<br>COPD: 5.2%<br>Previous cancer: 3.4%<br>Depression: 5.2%

© 2021 Nasserie T et al. *JAMA Network Open*.

Rosales-Castillo[31]	Spain	Non-concurrent, single-arm	Positive SARS-CoV-2 real-time reverse transcriptase PCR or serological test	Not reported				60.16 (15.08)	55.9	Hypertension: 50%<br>Hypercholesterolemia (28%)<br>Type 2 diabetes: 22%<br>Asthma: 14.4%<br>Sleep apnea-hypopnea syndrome: 8.5%<br>COPD: 6%<br>Ischemic heart disease: 6%<br>Chronic kidney disease: 6%
Shah[32]	Canada	Concurrent, single-arm	Positive SARS-CoV-2 real-time reverse transcriptase PCR	Not reported				Median: 67 (IQR: 54 to 74)	68.3	Median BMI: 25 (IQR: 23-29)<br>Hypertension: 35%<br>Diabetes: 22%<br>Chronic pulmonary disease: 13%<br>Coronary heart disease: 10%<br>Malignancy: 10%<br>Chronic kidney disease: 7%<br>Ever smoker: 38%
Sonnwebber, Eur Resp J[33]	Austria	Concurrent, single-arm	Positive SARS-CoV-2 real-time reverse transcriptase PCR and typical clinical presentation	0	50.3	27.6	22.1	57 (14)	54.9	None: 23%<br>Mean BMI (SD): 26 (5)<br>Current smoker: 3%<br>Past smoker: 39%<br>Hypertension: 30%<br>Diabetes mellitus: 17%<br>Cardiovascular disease: 40%<br>Chronic obstructive pulmonary disease: 6%<br>Asthma: 7%<br>Interstitial lung disease: 1%<br>Hypercholesterolemia: 19%<br>Chronic kidney disease: 7%<br>Chronic liver disease: 6%<br>Malignancy: 12%<br>Immunodeficiency: 6%
Sonnweber, Resp Res[34]	Austria	Concurrent, single-arm	Positive SARS-CoV-2 real-time	0	51	32	17	58 (14)	59.6	Hypertension: 29%<br>Diabetes mellitus: 18%<br>Cardiovascular disease: 40%

© 2021 Nasserie T et al. *JAMA Network Open*.

			reverse transcriptase PCR<br>Symptom presentation							Pulmonary disease: 19%<br>Endocrine disease: 45%<br>Chronic kidney disease: 6%<br>Chronic liver disease: 6%<br>None: 19%
Sykes[35]	England	Concurrent, single-arm	Positive SARS-CoV-2 real-time reverse transcriptase PCR	Not reported				59.6 (14.0)	65.7	Type 1 diabetes: 0.7%<br>Type 2 diabetes: 22.0%<br>Ischaemic heart disease: 16.4%<br>COPD: 8.2%<br>Asthma: 14.2%<br>Hypertension: 41.0%<br>CKD: 4.5%<br>History of venous thromboembolism: 2.2%<br>Cancer: 5.2%<br>Cardiovascular disease: 4.5%<br>Smoking history: 44.0%<br>Alcohol use: 42.5%
Taboada[36]	Spain	Concurrent, single-arm	Positive SARS-CoV-2 real-time reverse-transcriptase PCR	0	0	0	100	65.5 (10.4)	64.8	Hypertension: 55.5%<br>Hyperlipidemia: 44%<br>Diabetes: 23.1%<br>Asthma: 6.6%<br>COPD: 8.8%<br>Heart disease: 20.9%<br>Obesity: 38.5%
Tomasoni[37]	Italy	Concurrent, single-arm	Positive SARS-CoV-2 real-time reverse transcriptase PCR	Not reported				55 (range: 43 to 65)	73.3	Charlson Comorbidity Score, median (IQR): 1 (0 to 2.5)
Townsend[38]	Ireland	Concurrent, single-arm	Positive SARS-CoV-	Not reported				49.15 (15)	46.1	BMI (kg/m^2), mean (+/- SD): 28.7 +/- 5.3

© 2021 Nasserie T et al. *JAMA Network Open*.

			2 real-time reverse transcriptase PCR							Total number of comorbidities, median (IQR): 1 (0-2)
Ugurlu[39]	Turkey	Concurrent, single-arm	Positive SARS-CoV-2 real-time transcriptase PCR	Not reported				41.2 (14.6)	45.2	Not reported
Vaira[40]	Italy	Concurrent, single-arm	Positive SARS-CoV-2 real-time reverse transcriptase PCR	Not reported				51.2 (8.8)	49.3	Diabetes: 10.9%<br>BMI > 30 kg/m^2: 29%<br>Cardiovascular disorder: 26.8%<br>Pulmonary disorder: 15.2%
van den Borst[41]	Netherlands	Concurrent, single-arm	Positive SARS-CoV-2 real-time reverse transcriptase PCR or clinical diagnosis	0	62.9	21	16.1	59 (14)	59.7	Diabetes mellitus: 14%<br>Hypertension: 28%<br>Cardiovascular disease: 24%<br>Asthma: 10%<br>Chronic obstructive pulmonary disease: 6%<br>Other lung disease: 3%<br>Immunocompromised: 15%<br>Chronic kidney failure: 8%<br>Cancer: 20%
Weerahandi[42]	United States	Concurrent, single-arm	Lab-confirmed	0	0	100	0	62 (IQR: 50 to 67)	62.5	Hypertension: 60.25%<br>Diabetes: 36.65%<br>Chronic kidney disease: 8.07%<br>Cancer: 7.45%<br>Coronary artery disease: 9.32%<br>Heart failure: 4.97%<br>Hyperlipidemia: 46.58%<br>Asthma or COPD: 24.22%<br>BMI (kg/m^2):

© 2021 Nasserie T et al. *JAMA Network Open*.

										<25: 14.29%<br>25 to <30: 30.43%<br>30 to <40: 40.99%<br>>/ 40: 13.66%<br>Missing: 0.62%
Wong[43]	Canada	Concurrent, single-arm	Lab-confirmed	Not reported				62 (16)	64.1	Diabetes: 26%<br>Pulmonary disease: 8%<br>History of heart attack: 8%<br>None: 59%
Xiong[44]	China	Concurrent, two-arm	Not stated	0	61.5	33.5	5	Median: 52 (IQR: 95 - 102)	45.5	Hypertension: 15.2%<br>Diabetes: 7.4%<br>Chronic obstructive lung disease: 4.1%<br>Coronary heart disease: 3.3%<br>Chronic kidney disease: 2.2%<br>Carcinoma: 0.9%
Zhao[45]	China	Non-concurrent, single-arm	Positive SARS-CoV-2 real-time reverse transcriptase PCR	0	93	7	0	47.7 (15.5)	58.2	Diabetes mellitus: 3.64%<br>Hypertension: 10.91%<br>Cardiovascular disease: 3.64%<br>Smoking: 4%<br>Pulmonary diseases: 0%

© 2021 Nasserie T et al. *JAMA Network Open*.

eTable 4. Selection Criteria

Study	Source of Participants	Inclusion & Exclusion Criteria	No. of Reported Eligible Participants	Number Excluded & Reasons for Non-Participation	Final Sample Size (Proportion of Total Eligible)
Akter[1]	Hospitalized: 100% (% in non-ICU and ICU not indicated)	Inclusion: Patients with confirmed infection	Not reported	Not reported	734 (NR)
Arnold[2]	Hospitalized: 100% (% in non-ICU and ICU not indicated)	Inclusion: Hospitalized patients<br><br>Exclusion: <18 years old; inability to consent to study participation; nursing home residents; unwilling or unable to undergo chest CT or provide blood sample	131	N=21<br>Declined follow-up (N=18)<br>Unreachable (N=3)	110 (83.9%)
Carfi[3]	Hospitalized: 87.4% in non-ICU, 12.6% in ICU	Inclusion: Patients discharged from hospital after recovery (based on WHO guidelines)	157	N=14<br>Declined participation (N=14)	143 (91.1%)
Carvalho-Schneider[4]	Outpatient: 64.6%<br>Hospitalized: 35.4% (ICU patients excluded)	Inclusion: Patients with confirmed infection<br><br>Exclusion: <18 years old; ICU patients; residents of nursing homes or long-term care facilities;	174	N=44<br>Lost to follow-up (N=44)	130 (74.7%)

© 2021 Nasserie T et al. *JAMA Network Open*.

		patients transferred to other healthcare facilities			
Chen[5]	Hospitalized: 100% (% in non-ICU and ICU not indicated)	Not clearly stated	503	N=142<br>Lost to follow-up (N=131)<br>Provided incomplete data (N=11)	361 (71.8%)
Chiesa-Estomba[6]	Hospitalized and outpatient (numbers not provided)	Inclusion: Hospitalized patients or outpatients<br><br>Exclusion: Admitted to ICU; pre-existing olfactory or gustatory dysfunction	1, 222	N = 471<br>Incomplete follow-up data (N=362)<br>Unreachable (N=61)<br>Declined participation (N=48)	751 (61.5%)
Chopra[7]	Hospitalized: 100% (% in non-ICU and ICU not indicated)	Inclusion: Hospitalized patients alive 60 days after discharge<br><br>Exclusion: Residing in a health care or correctional facility	1167	N = 679<br>Reasons not reported	488 (41.8%)
D'Cruz[8]	Hospitalized: 65.5% in non-ICU, 35.5% in ICU	Inclusion: Patients with severe COVID-19 (hospital length of stay ≥48 h and maximum fraction of inspired oxygen ≥40% or ICU admission)<br><br>Exclusion: <18 years	143	N = 24<br>Unreachable or declined participation (N=24)	119 (83.2%)

© 2021 Nasserie T et al. *JAMA Network Open*.

		old; mild or moderate COVID-19; unwilling or unable to undergo CT scan			
Daher[9]	Hospitalized: 100% (% in non-ICU and ICU not indicated)	Inclusion: Patients with confirmed infection<br><br>Exclusion: patients with Acute Respiratory Distress Syndrome who needed mechanical ventilation in the ICU; unwilling or unable to undergo CT scan, echocardiography, or whole-body plethysmography or provide blood sample	Not reported	Not reported	33 (NR)
de Graaf[10]	Hospitalized: 100% (59% in non-ICU, 41% in ICU)	Inclusion: Patients discharged from hospital<br><br>Excluded: Patients transferred to hospice; re-admitted patients; <18 years old; unable or unwilling to undergo chest CT scan	98	N = 17<br>Declined follow-up due to lack of symptoms (N = 7)<br>Administrative errors (N=10)	81 (82.7%)
Garrigues[11]	Hospitalized: 80% in non-ICU, 20% in ICU	Inclusion: Hospitalized patients<br><br>Exclusion: Deceased, unreachable, demented,	192	N=72<br>Unreachable (N=69)<br>Declined participation (N=2)<br>Other (N=1)	120 (62.5%)

© 2021 Nasserie T et al. *JAMA Network Open*.

		bedridden, and non-French speaking patients; patients admitted directly to ICU			
Gherlone[12]	Hospitalized: 100% (75.4% in non-ICU, 24.6% in ICU)	Inclusions: patients admitted to emergency department<br><br>Exclusion: <17 years	Not reported	Not reported	122 (NR)
Gonzalez[13]	Hospitalized: 100% in ICU	Inclusion: Patients discharged from ICU; acute respiratory distress syndrome due to COVID-19<br><br>Exclusion: <18 years; transferred to another hospital during hospitalization; palliative care patients; severe mental illnesses; unable or unwilling to undergo CT scan	75	N = 13<br>Unreachable or refused participation (N=13)	62 (82.7%)
Halpin[14]	Hospitalized: 68% in non-ICU, 32% in ICU	Inclusion: Patients discharged from hospital; resides in Leeds Metropolitan District<br><br>Exclusion criteria: <18 years old; dementia, learning disability, cognitive or	158	N = 58<br>Unreachable (N=56)<br>Declined participation (N=2)	100 (63.3%)

© 2021 Nasserie T et al. *JAMA Network Open*.

		communication impairment			
Huang[15]	Hospitalized: 95.6% in non-ICU, 4.4% in ICU	Inclusion: Patients discharged from hospital<br><br>Exclusion: individuals with psychotic disorders, dementia; re-admitted to hospital due to underlying diseases; immobile or unable to move freely due to osteoarthropathy, stroke, or embolism; living outside of Wuhan or in nursing or welfare homes; unwilling or unable to undergo CT scan	2, 142	N = 409<br>Declined participation (N=347)<br>Unreachable (N=62)	1733 (80.9%)
Jacobs[16]	Hospitalized: 100% (% in ICU and non-ICU not stated)	Inclusion: Patients discharged from hospital<br><br>Exclusion: Hospital stay less than 3 days; non-English speakers; individuals with dementia or delirium	351	N = 168<br>Reasons not reported	183 (52.1%)
Lechien[17]	Outpatient: 100%	Inclusion: Patients with mild or moderate COVID-19 presenting with sudden loss of smell	95	N = 7<br>Did not complete study (N=7)	88 (92.6%)

© 2021 Nasserie T et al. *JAMA Network Open*.

Lerum[18]	Hospitalized: 85.4 in non-ICU, 14.6% in ICU	Inclusion: Patients discharged from hospital<br><br>Exclusion: <18 years old; admitted for less than 8 hours; live outside the hospitals' catchment areas; inability to provide informed consent; participation in WHO Solidarity trial; unable or unwilling to undergo CT scan	Not reported	Not reported	103 (NR)
Liang[19]	Hospitalized: 90.8% in non-ICU, 9.2% in ICU	Inclusion: Patients discharged from hospital after recovery<br><br>Exclusion: <18 years old; history of pulmonary resection; neurological or psychiatric disease; unwilling or unable to undergo CT scan or provide blood sample	134	N = 58<br>Unreachable (N=11)<br>Not nearby during study period (N=6)<br>Declined participation (N=33)<br>Did not complete study (N=8)	76 (56.7%)
Lu[20]	Hospitalized: 100% (% in non-ICU and ICU not indicated)	Inclusion: Patients who recovered from COVD-19Recovered COVID-19 patients<br><br>Exclusion: Unwilling or unable to undergo MRI scan	155	N=95<br>Reasons not reported	60 (38.7%)
Mandal[21]	Hospitalized: 85.5% in	Inclusion: Patients	878	N=494	384 (43.7%)

© 2021 Nasserie T et al. *JAMA Network Open*.

	non-ICU, 14.5% in ICU	with confirmed infection<br><br>Exclusion: Unwilling to undergo chest radiograph or provide blood sample		Follow-up not completed for logistic reasons (N=430)<br>Unreachable by phone (N=53)<br>Declined follow-up (N=11)	
Mazza[22]	Outpatient: 21.7%*<br>Hospitalized: 78.3% (% in non-ICU and ICU not indicated)<br>*Diagnosed in hospital ER	Inclusion: Patients with confirmed COVID diagnosed in the emergency department<br><br>Exclusion: <18 years old	402	N=176<br>Reasons not reported	226 (56.2%)
Mendez[23]	Hospitalized: 81% in non-ICU, 19% in ICU	Inclusion: Hospitalized patients<br><br>Exclusion: ≥ 85 years old or <18 years old; non-Spanish speaking; nursing home residents; pre-existing dementia or cognitive decline or brain injury; current alcohol or substance use disorder (except nicotine); history of major psychiatric disorders	216	N = 37<br><br>Declined participation (N=5)<br>Unreachable (N=18)<br>Withdrew consent (N=9)<br>Did not complete study (N=5)	179 (82.9%)
Moreno-Perez[24]	Hospitalized: 91.3% in non-ICU, 8.7% in ICU	Inclusion: Hospitalized patients<br><br>Exclusion: Severe comorbidities	326	N = 49<br>Declined participation (N=4)<br>Did not attend face-to-face assessment (N=15)	277 (85.0%)

© 2021 Nasserie T et al. *JAMA Network Open*.

				Lost to follow-up (N=30)	
Munro[25]	Hospitalized: 98.3% in non-ICU, 1.7% in ICU	Inclusion: Patients discharged from hospital	Not reported	Not reported	121 (NR)
Nguyen[26]	Outpatient: 100%	Inclusion: Patients who reported anosmia and/or ageusia in the acute phase of infection	200	N=75<br>Lost to follow-up (N=75)	125 (62.5%)
Poncet-Megemont[27]	Outpatient: 54.7%<br>Hospitalized: 41% non-ICU, 4.3% ICU	Inclusion: Patients with confirmed infection	161	N=22<br>Declined participation (N=5)<br>Unreachable (N=17)	139 (86.3%)
Puntmann[28]	Outpatient: 67%<br>Hospitalized: 33% (% in non-ICU and ICU not indicated)	Inclusion: Minimum of 2 weeks from original diagnosis; resolution of respiratory symptoms; negative result on COVID-19 swab test<br><br>Exclusion: Unwilling or unable to take MRI scan	Not reported	Not reported	100 (NR)
Qu[29]	Hospitalized: 100% (% in non-ICU and ICU not indicated)	Inclusion: Patients discharged from hospital<br><br>Exclusion: Patients transferred to another medical facility for treatment of concurrent issues;	573	N = 33<br>Declined participation (N=14)<br>Did not complete study (N=19)	540 (94.2%)

© 2021 Nasserie T et al. *JAMA Network Open*.

		confirmed hepatitis B, C, AIDS or other viral infections; pregnant women			
Raman[30]	Hospitalized: 64% in non-ICU, 36% in ICU	Inclusion: patients with moderate to severe confirmed infection<br><br>Exclusion: unable or unwilling to undergo MRI; severe comorbidities	Not reported	Not reported	58 (NR)
Rosales-Castillo[31]	Hospitalized: 92.4% in non-ICU, 7.6% in ICU	Inclusion: Patients discharged from hospital	Not reported	Not reported	118 (NR)
Shah[32]	Hospitalized: 100% (% in non-ICU and ICU not reported)	Inclusion: Hospitalized patients<br><br>Exclusion: Unwilling or unable to undergo CT scan	82	N = 22<br>Declined participation (N=10)<br>Unreachable (N=7)<br>Did not complete study (N=5)	60 (73.2%)
Sonnwebber, Eur Resp J[33]	Outpatient: 25.6%<br>Hospitalized: 52.6% in non-ICU, 21.8% in ICU	Inclusion: Hospitalized patients or outpatients with persisting symptoms	190	N = 57<br>Unable to participate (N=27)<br>Unavailable for follow up (N=12)<br>Declined participation (N=18)	133 (70 %)
Sonnweber, Resp Res[34]	Outpatient: 20.2%<br>Hospitalized: 63.3% in non-ICU, 16.5% in ICU	Inclusion: Patients with confirmed infection<br><br>Exclusion: Unwilling	186	N=77<br>Lost to follow-up (N=59)<br>Declined participation (N=18)	109 (58.6%)

© 2021 Nasserie T et al. *JAMA Network Open*.

		to have blood sample taken or to undergo CT scan			
Sykes[35]	Hospitalized: 100% (79.9% in non-ICU, 20.1% in ICU)	Inclusion: Patients with confirmed infection treated for COVID-19 pneumonia<br><br>Exclusion: care home residents; Clinical Frailty Score ≥ 6;	190	N=56<br>Lost to follow-up (N=56)	134 (70.5%)
Taboada[36]	Hospitalized: 100% (100% in ICU)	Inclusion: Patients with COVID-19 induced acute respiratory distress syndrome requiring treatment in an ICU	92	N = 1<br>Declined participation (N=1)	91 (98.9%)
Tomasoni[37]	Hospitalized: 100% (% in non-ICU and ICU not indicated)	Inclusion: Patients who recovered from COVID-19<br>Recovered patients COVID-19 patients	Not reported	Not reported	105 (NR)
Townsend[38]	Outpatient: 44.5%<br>Hospitalized: 41.4% in non-ICU, 14.1% in ICU	Inclusion: Minimum of 6 weeks after date of last acute COVID-19 symptoms for outpatients or date of discharge for patients admitted to hospital during illness	223	N=95<br>Reasons not reported	128 (57.4%)
Ugurlu[39]	Hospitalized: 100% (ICU patients	Inclusion: Hospitalized patients	42	N=0	42 (100%)

© 2021 Nasserie T et al. *JAMA Network Open*.

	excluded)	with olfactory dysfunction<br><br>Exclusion: <18 years of age or >60 years of age; history of nasal surgery or olfactory dysfunction; chronic sinusitis; neurological or psychiatric diseases			
Vaira[40]	Outpatient: 77%<br>Hospitalized: 23% non-ICU (*ICU patients excluded)	Inclusion: severe COVID-19 diagnosis; symptomatic patients presenting within 4 days of symptom onset<br><br>Exclusion: <18 years old; admitted to ICU; patients with history of previous trauma, surgery, or radiotherapy in oral and nasal cavities; patients with allergic rhinitis or rhinosinusitis; patients with mental health illnesses	146	N=8<br>Lost to follow-up (N=8)	138 (94.5%)
van den Borst[41]	Outpatient: 21.8%<br>Hospitalized: 62.1% in non-ICU, 16.1% in ICU	Inclusion: Hospitalized patients, or patients referred by general practitioners<br><br>Exclusion: unable or	197	N = 73<br>Declined participation (N=69)<br>Unreachable (N=2)<br>Unknown (N=2)	124 (62.9%)

© 2021 Nasserie T et al. *JAMA Network Open*.

		unwilling to undergo chest CT (hospitalized patients) or to provide blood sample			
Weerahandi[42]	Hospitalized: 53.9% in non-ICU, 46.1% in ICU	Inclusion: Hospitalized patients needing at least 6L of oxygen at any point during hospitalization<br><br>Exclusion: <18 years old; deceased, demented, or communication impaired patients; patients discharged to hospice; residents of long-term care facilities prior; patients fully dependent in daily living activities prior to hospitalization; re-hospitalized	390	N=238<br>Unreachable (N=135)<br>Declined participation (N=94)<br>Lost to follow-up (N=9)	152 (39.0%)
Wong[43]	Hospitalized: 100% (% in non-ICU and ICU not indicated)	Inclusion: Hospitalized patients<br><br>Exclusion: <18 years old; inability to complete surveys in English	96	N=18<br>Declined participation (N=10)<br>Could not complete questionnaires because seen virtually (N=8)	78 (81.3%)
Xiong[44]	Hospitalized: 100% (% in non-ICU and ICU not stated)	Inclusion: Patients discharged from hospital	706	N = 168<br>Unreachable (N=87)<br>Declined participation (N=75)	538 (76.2%)

© 2021 Nasserie T et al. *JAMA Network Open*.

		Exclusion: <20 years old or >80 years old; pregnant or lactating patients; transferred to another hospital for treatment; recent surgery or chemotherapy; presence of other serious disease; incomplete medical record		Inability to describe symptoms clearly (N=6)	
Zhao[45]	Hospitalized: 100% (*ICU patients excluded)	Inclusion: Patients with confirmed infection<br><br>Exclusion: <18 years old; ICU patients; unable or unwilling to undergo chest CT	73	N=18<br>Unreachable (N=4)<br>Declined participation (N=14)	55 (75.3%)

© 2021 Nasserie T et al. *JAMA Network Open*.

eTable 5. Follow-Up and Outcome Measurement

Study	Time Zero	Length of Final Follow-Up	Determination of End of Follow-Up	Outcome Measurement
Akter[1]	Recovery	4 weeks after recovery	All participants followed for same amount of time	Self-report
Arnold[2]	Hospital admission	8 to 12 weeks after admission<br>Minimum: 56 days	Varied for each participant; based on date of examination	X-ray (chest abnormalities); blood sample (lab assessments); SF-36 Survey (QOL); self-report (other outcomes)
Carfi[3]	Symptom onset	Mean: 60.3 days after symptom onset (SD: 13.6) or 36.1 days after hospital discharge<br>Minimum: not stated	Varied for each participant; based on date of examination	EQ-VAS (QOL); patient reporting survey (other outcomes)
Carvalho-Schneider[4]	Symptom onset	Mean: 59.7 days after symptom onset (range: 57 to 67 days)<br>Minimum: 57 days	Varied for each participant, although intended to be at day 60; based on date of examination	mMRC Dyspnea Scale (dyspnea); 10-point analog scale (chest pain, anosmia, ageusia)
Chen[5]	Hospital discharge	1 month after hospital discharge	All participants followed for same amount of time	SF-36 (QOL)
Chiesa-Estomba[6]	Recovery	Mean 47 (IQR:30,71) after first consultation<br>Minimum of 30 days after negative test	Varied for each participant; based on date of examination	sQOD-NS (olfactory function)
Chopra[7]	Hospital discharge	Sixty days after hospital discharge	All participants followed for same amount of time	Self-report
D'Cruz[8]	Hospital discharge	61 days after hospital discharge (IQR: 51-67)	Varied for each participant; based on date of examination	mMRC Dyspnea Scale (dyspnea); PHQ-9 (depression); TSQ (trauma); GAD-7 (anxiety); 6-CIT (cognitive impairment); CT scans (organ functioning); 4MGS (gait speed); 1-minute Sit to Stand Test (mobility)
Daher[9]	Hospital discharge	6 weeks after discharge	All participants followed for same amount of time	PHQ-9 (depression); GAD-7 (anxiety); EQ-5D-5L (QOL); 6 minute walk test (mobility); blood sample (lab assessments); electrocardiography, CT scans (organ functioning)
de Graaf[10]	Hospital discharge	6 weeks after hospital discharge	All participants followed for the same amount of time	CT scans (organ functioning); pulmonary function tests; GAD-7 (anxiety); PHQ-9 (depression); PCL_5 (PTSD); CFQ-25 (cognitive impairment); IQ-CODE-N (cognitive impairment among elderly patients); NYHA (dyspnea)
Garrigues[11]	Hospital admission	Mean: 110.9 days after admission<br>Minimum: 100 days	Varied for each participant; based on date of examination	mMRC Dyspnea Scale (dyspnea); EQ-5D-5L (QOL); self-report (other outcomes)
Gherlone[12]	Hospital discharge	Median: 104 days after hospital discharge (IQR: 95 to 132)	Varied for each participant; based on date of examination	Extraoral and intraoral physical examination (facial abnormalities)
Gonzalez[13]	Hospital discharge	3 months after hospital discharge	All participants followed for the same amount of time	SF-12 (QOL); HADS (depression); CT scan (organ functioning); mMRC Dyspnea scale (dyspnea); pulmonary function test
Halpin[14]	Hospital discharge	Mean: 48 days(Range: 29,71) (SD: 17 days)	Varied for each participant; based on date of examination	EQ-5D-5L (QOL); Telephone screening tool (all other symptoms)
Huang[15]	Symptom onset	Median: 186 days after symptom onset (IQR: 175 to 199)	Varied for each participant; based on date of examination	mMRC (dyspnea); EQ-5D-5L (QOL, anxiety, depression); EQ-VAS (QOL); blood sample (lab assessments); CT scans (organ function); 6-min walk test (mobility)
Jacobs[16]	Hospital discharge	Mean: 35 day after	Varied for each participant;	PROMIS (all outcomes)

© 2021 Nasserie T et al. *JAMA Network Open.*

		discharge (range: 30 to 40 days)	based on date of examination	
Lechien[17]	Diagnosis	Two months	All participants followed for the same amount of time	SNOT-22 (sinonasal outcomes); sQOD-ns (olfactory function); NHANES (olfactory and gustatory function); 16-item Sniffiin-Sticks identification test (psychosocial olfactory evaluation)
Lerum[18]	Hospital admission	Median: 83 days after hospital admission (IQR: 73 to 90 days)	Varied for each participant; based on date of examination	mMRC Dyspnea Scale (dyspnea); EQ-5D-5L (QOL); chest CT scan (organ function);
Liang[19]	Hospital discharge	3 months	All participants followed for same amount of time	Spirometry (pulmonary function); CT scans (organ functioning); blood sample (lab assessments)
Lu[20]	Diagnosis	Three months after diagnosis	All participants followed for same amount of time	MRI scan (cerebral activity); self-report (other outcomes)
Mandal[21]	Hospital discharge	Median: 54 days after discharge ((IQR 47–59))	Varied for each participant; based on date of examination	X-ray (chest abnormalities); blood sample (lab assessments); PHQ-2 (depression); self-report (other outcomes)
Mazza[22]	Hospital discharge	90.1 days after hospital discharge (SD: 13.4)	Varied for each participant; based on date of examination	IES-R (distress); PCL-5 (PTSD); ZSDS (depression); BDI-13 (depression); STAI-Y (anxiety); WHIIRS (insomnia); OCI (obsessive compulsive disorder); BACS (cognitive function); clinical charts (inflammatory markers)
Mendez[23]	Hospital discharge	2 months (±1 month) after hospital discharge	Varied for each participant; based on date of examination	SF-12 (QOL); SCIP (verbal memory); ANT (verbal fluency); WAIS-III (working memory); GAD-7 (anxiety); PHQ-2 (depression); DTS (PTSD)
Moreno-Perez[24]	Symptom onset	Median: 76 days (IQR: 72 to 83 days)	Varied for each participant; based on date of final examination	EQ-VAS (QOL); x-ray (chest abnormalities); blood sample (lab assessments); pulmonary function test
Munro[25]	Hospital discharge	8 weeks	All participants followed for the same amount of time	General questionnaire
Nguyen[26]	Symptom onset	Mean: 221.7 days after symptom onset (SD: 10.9, range: 201-234)	Varied for each participant, based on date of final examination	Self-report
Poncet-Megemont[27]	Recovery	Mean: 1 month after recovery (range: 30-35 days)<br>Minimum: 30 days	Varied for each participant; based on date of examination	Self-report
Puntmann[28]	Diagnosis	Median: 71 days after diagnosis (IQR: 64 to 92 days)<br>Minimum: not stated	Varied for each participant; based on date of examination	MRI scan (cardiac activity); self-report (other outcomes)
Qu[29]	Hospital discharge	3 months after hospital discharge	All participants followed for the same amount of time	SF-36 (QOL); self-report (all other symptoms)
Raman[30]	Symptom onset	Median: 2 to 3 months after symptom onset (IQR: 2.05 to 2.53 months)	Varied for each participant; based on date of examination	MRI scan (organ activity); spirometry (lung functioning); 6 minute walk test (mobility); PHQ-9 (depression); GAD-7 (anxiety); MoCA (cognitive functioning); mMRC Dyspnea Scale (dyspnea); FSS (fatigue); SF-36 (QOL)
Rosales-Castillo[31]	Hospital discharge	50.8 days after hospital discharge (SD: 6.02)	Varied for each participant, based on date of final examination	Self-report
Shah[32]	Symptom onset	Mean: 11.7 weeks after symptom onset (range:<br>8 to 12 weeks)	Varied for each participant; based on date of examination	Detailed pulmonary function testing (pulmonary function); 6 minute walk test (mobility); CT scans (organ functioning)
Sonnwebber, Eur Resp J[33]	Diagnosis	Mean: 103 days after diagnosis (SD: 21)	Varied for each participant; based on date of examination	mMRC Dyspnea Scale (dyspnea); spirometry, blood plethysmography (pulmonary

© 2021 Nasserie T et al. *JAMA Network Open.*

				function); chest CT scan (organ function); blood sample (lab assessments); trans-thoracic echocardiography (cardiac function)
Sonnweber, Resp Res[34]	Symptom onset	60 days (SD ± 12) after symptom onset	Varied for each participant, although intended to be at day 60; based on date of examination	6-minute walk test (mobility); CT scan (lung functioning); blood sample (lab assessments)
Sykes[35]	Hospital discharge	Median: 113 days post-discharge, range: 46-167 days	Varied for each participant; based on date of examination	X-ray (chest abnormalities; mMRC Dyspnea Scale (dyspnea); EQ-5D-5L (QOL); direct questioning (all other symptoms)
Taboada[36]	Hospital discharge	6 months after hospital discharge	All participants followed for the same amount of time	EQ-5D-5L (QOL); PCFS (functional status)
Tomasoni[37]	Recovery	Median: 46 days after recovery (IQR: 43 to 48 days)<br>Minimum: 30 days	Varied for each participant; based on date of examination	HADS (anxiety and depression); MMSE (cognitive disorders)
Townsend[38]	Hospital discharge and diagnosis	Median: 72 days after hospital discharge for hospitalized patients or timepoint of 14 days following diagnosis for outpatients (IQR: 62 to 87 days)<br>Minimum: 42 days	Varied for each participant; based on date of examination	CFQ-11 (fatigue)
Ugurlu[39]	Hospital admission	Three months	All participants followed for the same amount of time	BSIT (olfactory function)
Vaira[40]	Symptom onset	60 days after initial symptom onset	All participants followed for same amount of time	Self-administered olfactory and gustatory psychosocial tests (anosmia and ageusia/dysgeusia, outpatients); CCCRC Olfactory Test (anosmia and ageusia/dysgeusia, hospitalized patients)
van den Borst[41]	Hospital discharge and symptom onset	Mean of 10 weeks (SD: 1.7 weeks) since hospital discharge (hospitalized patients - 78.2% of participants)<br>Mean of 14.7 weeks (SD: 2.2 weeks) since symptom onset for referred patients (21.8% of participants)	Varied for each participant; based on date of examination	Resting pulse-oximetry, spirometry (pulmonary functioning); mMRC Dyspnea Scale (dyspnea); CT scan and x-ray (chest functioning); CFS (frailty); HADS (anxiety and depression); TICS, CFQ (cognitive functioning); PCL-5 and IES-R (PTSS); SF-36 (QOL); blood sample (lab assessments)
Weerahandi[42]	Hospital discharge	Median: 37 days (range: 30 to 43 days) after hospital discharge<br>Minimum: 30 days	Varied for each participant; based on date of examination	PROMIS (all outcomes)
Wong[43]	Symptom onset	Median: 13 weeks after symptom onset (IQR: 11-14 weeks)	Varied for each participant; based on date of examination	EQ-5D-5L (QOL); Frailty Index, UCSD (frailty); SOB Questionnaire, UCSD (shortness of breath); Pittsburgh Sleep Quality Index (sleep quality); PHQ-9 (depression)
Xiong[44]	Hospital discharge	Median: 97 days after hospital discharge (range: 95 to 102 days)	Varied for each participant; based on date of examination	Self-report
Zhao[45]	Hospital discharge	Range: 64 to 93 days after hospital discharge<br>Minimum: 64 days	Varied for each participant; based on date of examination	Medical records; CT scan (chest functioning); spirometry (pulmonary functioning); self-report (other outcomes)

© 2021 Nasserie T et al. *JAMA Network Open*.

eTable 6. Reported Outcomes and Frequencies at Follow-Up

Study	Akter[1]	Arnold[2]	Carfi[3]	Carvalho-Schneider[4]	Chen[5]	Chiesa-Estomba[6]	Chopra[7]	D'Cruz[8]	Daher[9]	de Graaf[10]	Garrigues[11]	Gherlone[12]	Gonzalez[13]	Halpin[14]	Huang[15]	Jacobs[16]	Lechien[17]	Lerum[18]	Liang[19]	Lu[20]	Mandal[21]	Mazza[22]	Mendez[23]	Moreno-Perez[24]	Munro[25]	Nguyen[26]	Poncet-Megemont[27]	Puntmann[28]	Qu[29]	Raman[30]	Rosales-Castillo[31]	Shah[32]	Sonnweber, Resp Res[34]	Sonnweber, Eur Resp J[33]	Sykes[35]	Taboada[36]	Tomasoni[37]	Townsend[38]	Ugurlu[39]	Vaira[40]	van den Borst[41]	Weerahandi[42]	Wong[43]	Xiong[44]	Zhao[45]
**Sample Size**	734	110	143	130	361	751	488	119	33	81	120	122	62	100	1733	183	88	103	76	60	384	226	179	277	121	125	139	100	540	58	118	60	109	145	134	91	105	128	42	138	124	152	78	538	55
**Interpretation: % of study sample continuing to experience outcome at the end of the individual or study follow-up period unless indicated otherwise**																																													
**Symptom Persistence (≥ 1 symptom at follow-up)**		74%	87%	66%				89%					73%		76%						72%			50.9%					57.6%		62.5%			41%	86%	84%	52.4%						76%	49.6%	
**Cardiac**																																													
**Atypical chest pain**		12.7%†	21.7%	13.1%					18%	18%	10.8%				2.9%													17%							17.9%	8.8%								12.3%	
**Palpitations**				10.9%						14%					8.9%				62%									20%	20.4%																
**Chest tightness**																			62%																										
**Chest distress**																																												14.1%	
**Resting heart rate increase**																																												11.2%	

© 2021 Nasserie T et al. *JAMA Network Open*.

**Discontinuous flushing**																																												4.8%	
**Cardiac Magnetic Resonance Imaging**																																													
**Myocardial inflammation (abnormal native T1 and T2)**																												60%																	
**Myocardial LGE**																												32%																	
**Non-ischemic LGE**																												20%																	
**Ischemic LGE**																												12%																	
**Pericardial LGE**																												22%																	
**Pericardial effusion**																												20%																	
**Neurological**																																													
**Memory loss/impairment**	19.2%										34.2%			18%						28.3%															37.3%										
**Immediate verbal memory impairment**																						10%	49.2%																						
**Delayed memory impairment**																							14.6%																						
**Semantic verbal fluency impairment**																						32%	43%																						
**Working memory impairment**																						24%	7.2%																						
**Attention and information processing impairment**																						33%																							
**Psychomotor coordination impairment**																						57%																							
**Executive functions impairment**																						50%																							

© 2021 Nasserie T et al. *JAMA Network Open*.

**Concentration issues**	25.6%										28%			22%																					25.4%										
**Confusion**																8.74%																													
**Cognitive deficit**								21.6%	18%	27%																									9.7%		17.1%				15%				
**Mood change/low mood**																				16.7%															39.6%										
**Anxiety**								22.1%		5%			22.1%									30%	29.6%							14%					47.8%		29%				10%			6.5%	
**Depression**								18%		17%			15.2%								14.6%	9%	26.8%							19%							11%				12%			4.3%	
**Anxiety or depression**	21.6%													23%	21.1%																												14.3%		
**Panic attack**	13.4%																																												
**Fatigue/exhaustion/asthenia**		39.1%†	53.1%	40%				68%	45%		55%		29.5%	64%	59.9%	44.8%			59%	26.7%	69%			34.8%				36%	29.4%	55%	30.5%				39.6%	37.4%	31.4%	52.3%			69%			28.3%	16.4%
**PTSD**								24.8%						31%								13%	25.1%																						
**Dysphoria**																																												1.7%	
**Respiratory**																																													
**Shortness of breath/dyspnea**		39.1%†	43.4%	30%					33%	62%	41.7%			50%		31.7%				-	53%			34.4%				36%	26.1%		31.4%	20%				57.1%	27.6%					74.3%	50%		14.6%

© 2021 Nasserie T et al. *JAMA Network Open*.

**mMRC ≥ 0**															69.0%			54%																											
**mMRC ≥ 1******													46.7%		24.2%			19%																											
**mMRC ≥ 2****				7.7%							29%																			64%															
**mMRC unspecified**								44%																										36%	9.7%										
**Cough**		11.8%†					15.4%	42.6%	33%		16.7%		16.4%	16.2%		25.1%			60%		34%			21.3%					11.7%		5%	20%		17%	35.1%	14.4%							23%	7.1%	
**Rhinorrhea**									12%																																				
**Hemoptysis**									0%																																				
**Sore throat**									9%						4%																				12.7%									3.2%	
**Post-activity polypnea**																																												21.4%	
**Nonmotor polypnea**																																												4.7%	
**Lung Imaging**																																													
**Abnormal chest radiograph appearance**																					38%																								
**Deteriorating chest radiograph appearance**																					9%																								
**Consolidation**		1%																																											
**Reticulation**		7%																																											
**Atelectasis**		5%																																											

© 2021 Nasserie T et al. *JAMA Network Open*.

**Pleural effusion**		1%																																											
**Neuromuscular**																																													
**Myalgia**		22.7%†							15%						2.3%					25%											13%					37.4%								4.5%	
**Arthralgia**		4.5%†		16.3%																																28.6%								7.6%	
**Myalgia or arthralgia**																								19.6%											51.5%										
**Impaired mobility**	16.9%													37%	6.5%					6.7%																56%									
**Numbness in extremities**																				6.7%																									
**Dysbasia**																													.4.1%																
**Tremor**																				1.7%																									
**Joint pain**			27.3%												8.9%	15.8%													26.3%																
**Muscle pain**																21.3%																													
**Headache**		1.82%†							15%						1.9%	12.6%				10%							3.6%																		18.18%
**Chills**																																												4.6%	

© 2021 Nasserie T et al. *JAMA Network Open*.

**Limb oedema**																																												2.6%	
**Dizziness**															5.8%																													2.6%	
**Trigeminal neuralgia**												2%																																	
**Gastrointestinal**																																													
**Diarrhea**		0.91%†		3.85%					9%							3.8%			26%					10.5%					6.7%																
**Diarrhea or vomiting**															4.6%																			9%											
**Abdominal or stomach pain**		1.82%†							3%																																				
**Nausea**		0%†							6%																																				
**Appetite issues**														8%	8%																														
**Quality of Life**																																													
**Returned to work**							60%				67.9%			20%																								69%							100%
**Worsened self-care**	9.6%													16%																						13%									
**Resumed sports**											71.8%																																		
**Physical health (composite)**		40.2*†			55.9 (7.24)*								45.9 (36.1 to 54																													43.8 (9.3)***			

© 2021 Nasserie T et al. *JAMA Network Open*.

												.4)																																
**Mental health (composite)**		44.8*†			48.92 (10.81)*								55.8 (40.6 to 58)																													47.3 (9.3)***			
**Poor or fair physical health**																27.1%							44.1%						15.4%																
**Poor or fair mental health**																16.9%							39.1%						32.6%																
**Pain/discomfort**								49.6%						19%	24.9%																			24%		48%									
**Circulatory and Immune System**																																													
**Iron deficiency**																																	30%												
**Anemia**																																	9.2%												
**Hyperferritinemia**																																	38%												
**Elevated IL6 (inflammation marker)**									0%																								12%												
**Elevated CRP (inflammation marker)**		1.8%																			9.5%%												16%												
**Elevated d-dimer (blood clot marker)**																					30.1%																								
**Lymphopenia**		1.8%																			7.9%																								

© 2021 Nasserie T et al. *JAMA Network Open*.

**Other**																																													
**Visual loss**																								5.4%																					
**Eye irritation**																8.20%																													
**Phlegm**																14.8%																													
**Pharyngalgia**									0%																																				
**Ageusia/Dysgeusia**									33.3%		10.8%	17%	0%		6.9%	22.8%				25%						11.5%	19.8%				2%				9.0%		9.5%			10.7*					
**Anosmia**		11.8%†				37%			50%		13.3%	14%	1.6%		10.2%	26.2%	20.5%			100%						23.3%	23.8%				4%			44.4%	9.7%	11.0%	10.5%		14.3%	9.5%					
**Hair loss**	9.7%										20%				20.7%																													28.6%	
**Sleep disorders/insomnia**		23.6%†						56.5%			30.8%				25.2%							3.1%												28%	35.1%	30.8%									
**Night sweats**																																		24%											
**Somnipathy**																																												17.7%	
**Fever**		0.91%†		0%					3%				1.6%			1.09%			20%					0%										0%	10.4%		0%								
**Ulcer**																1.09%																													

© 2021 Nasserie T et al. *JAMA Network Open*.

**Salivary gland ectasia**												38%																																	
**Dry mouth**												30%																																	
**TMJ abnormalities**												9%																																	
**Masticatory muscle weakness**												19%																																	
**Facial tingling**												3%																																	
**Facial asymmetry**												0.8%																																	
**Burning Pain**																																					10.5%								
**Vision change**																				1.7%																									
**Hearing loss**																				1.67%					6.6%																				
**Tinnitus**																									6.6%																				
**Difficulty swallowing**														8%																															
**Laryngeal sensitivity**														17%																															
**Voice change**														20%																															
**Difficulty communicating**														6%																															
**Bowel control issues**														3%																															
**Bladder control issues**														10%																															

* 36 item short-fom survey (SF-36): lower scores indicate higher disability, score of 100 represents the best possible health status. Values presented represent mean (SD).
** mMRC ≥ 2: Walks slower than people of the same age because of dyspnoea or has to stop for breath when walking at own pace
*** Patient-Reported Outcomes Measurement Information System 10-Question Short-Form (PROMIS-10 Global Health): Converted normed t-scores, where mean score of 50 and SD of 10 represents the mean for the general US population
†weighted average of mild, moderate, and severe baseline COVID-19 patient groups

© 2021 Nasserie T et al. *JAMA Network Open*.

eFigure. MOOSE Flowchart for the Literature Search

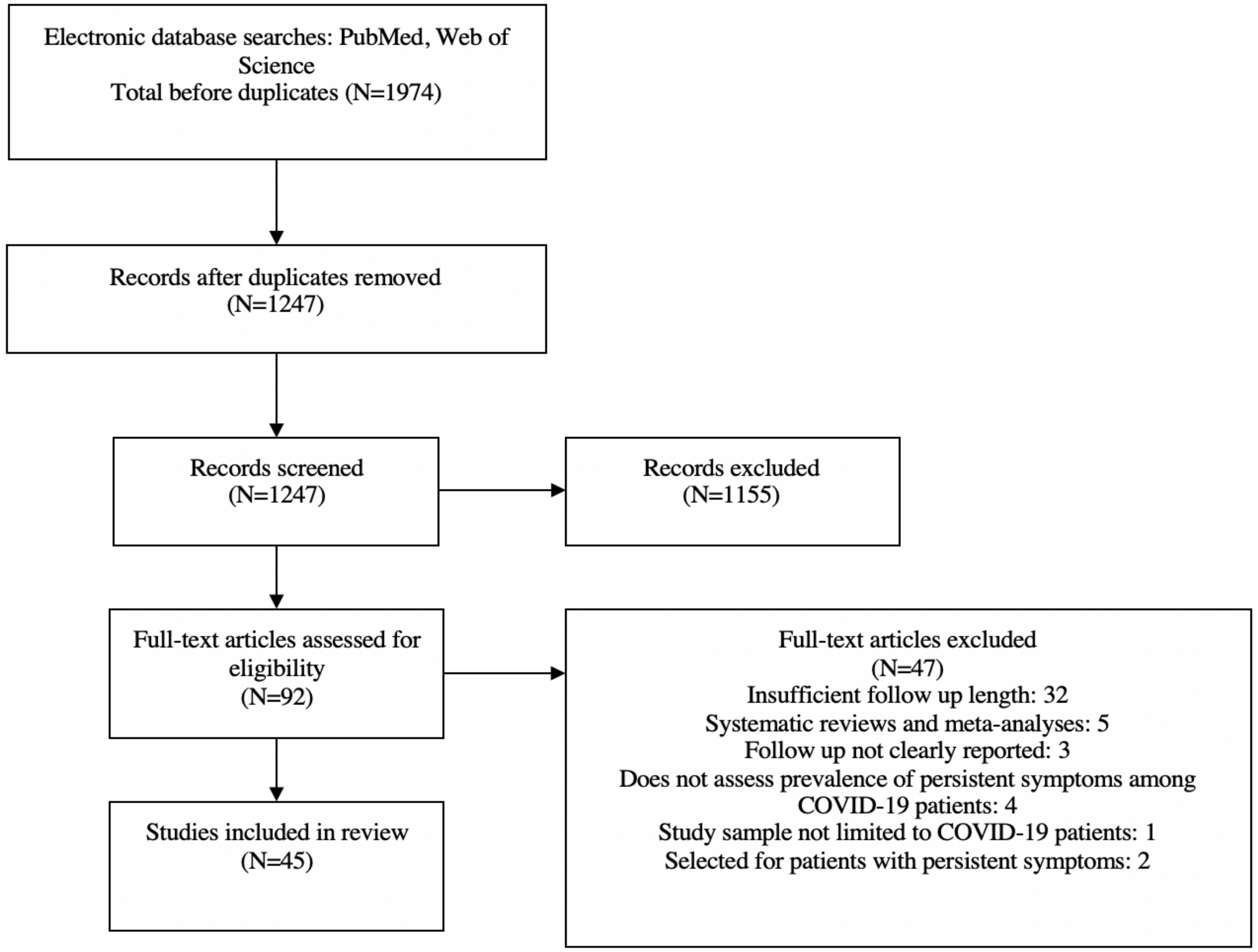

© 2021 Nasserie T et al. *JAMA Network Open*.

eReferences

1. Akter F, Mannan A, Mehedi HMH, et al. Clinical characteristics and short term outcomes after recovery from COVID-19 in patients with and without diabetes in Bangladesh. *Diabetes Metab Syndr*. 2020;14(6):2031-2038. doi:10.1016/j.dsx.2020.10.016

2. Arnold DT, Hamilton FW, Milne A, et al. Patient outcomes after hospitalisation with COVID-19 and implications for follow-up: results from a prospective UK cohort. *Thorax*. Published online December 3, 2020. doi:10.1136/thoraxjnl-2020-216086

3. Carfì A, Bernabei R, Landi F, Gemelli Against COVID-19 Post-Acute Care Study Group. Persistent Symptoms in Patients After Acute COVID-19. *JAMA*. 2020;324(6):603-605. doi:10.1001/jama.2020.12603

4. Carvalho-Schneider C, Laurent E, Lemaignen A, et al. Follow-up of adults with non-critical COVID-19 two months after symptoms' onset. *Clin Microbiol Infect Off Publ Eur Soc Clin Microbiol Infect Dis*. Published online October 5, 2020. doi:10.1016/j.cmi.2020.09.052

5. Chen K-Y, Li T, Gong F-H, Zhang J-S, Li X-K. Predictors of Health-Related Quality of Life and Influencing Factors for COVID-19 Patients, a Follow-Up at One Month. *Front Psychiatry*. 2020;11:668. doi:10.3389/fpsyt.2020.00668

6. Chiesa-Estomba CM, Lechien JR, Radulesco T, et al. Patterns of smell recovery in 751 patients affected by the COVID-19 outbreak. *Eur J Neurol*. 2020;27(11):2318-2321. doi:10.1111/ene.14440

7. Chopra V, Flanders SA, O'Malley M, Malani AN, Prescott HC. Sixty-Day Outcomes Among Patients Hospitalized With COVID-19. *Ann Intern Med*. Published online November 11, 2020. doi:10.7326/M20-5661

8. D'Cruz RF, Waller MD, Perrin F, et al. Chest radiography is a poor predictor of respiratory symptoms and functional impairment in survivors of severe COVID-19 pneumonia. *ERJ Open Res*. 2021;7(1). doi:10.1183/23120541.00655-2020

9. Daher A, Balfanz P, Cornelissen C, et al. Follow up of patients with severe coronavirus disease 2019 (COVID-19): Pulmonary and extrapulmonary disease sequelae. *Respir Med*. 2020;174:106197. doi:10.1016/j.rmed.2020.106197

10. de Graaf MA, Antoni ML, Ter Kuile MM, et al. Short-term outpatient follow-up of COVID-19 patients: A multidisciplinary approach. *EClinicalMedicine*. 2021;32:100731. doi:10.1016/j.eclinm.2021.100731

11. Garrigues E, Janvier P, Kherabi Y, et al. Post-discharge persistent symptoms and health-related quality of life after hospitalization for COVID-19. *J Infect*. Published online August 25, 2020. doi:10.1016/j.jinf.2020.08.029

12. Gherlone EF, Polizzi E, Tetè G, et al. Frequent and Persistent Salivary Gland Ectasia and Oral Disease After COVID-19. *J Dent Res*. Published online March 3, 2021:22034521997112. doi:10.1177/0022034521997112

13. González J, Benítez ID, Carmona P, et al. Pulmonary function and radiological features in survivors of critical COVID-19: a 3-month prospective cohort. Published online March 4, 2021. doi:10.1016/j.chest.2021.02.062

14. Halpin SJ, McIvor C, Whyatt G, et al. Postdischarge symptoms and rehabilitation needs in survivors of COVID-19 infection: A cross-sectional evaluation. *J Med Virol*. 2021;93(2):1013-1022. doi:10.1002/jmv.26368

15. Huang C, Huang L, Wang Y, et al. 6-month consequences of COVID-19 in patients discharged from hospital: a cohort study. *Lancet Lond Engl*. 2021;397(10270):220-232. doi:10.1016/S0140-6736(20)32656-8

16. Jacobs LG, Gourna Paleoudis E, Lesky-Di Bari D, et al. Persistence of symptoms and quality of life at 35 days after hospitalization for COVID-19 infection. *PloS One*. 2020;15(12):e0243882. doi:10.1371/journal.pone.0243882

17. Lechien JR, Journe F, Hans S, et al. Severity of Anosmia as an Early Symptom of COVID-19 Infection May Predict Lasting Loss of Smell. *Front Med*. 2020;7:582802. doi:10.3389/fmed.2020.582802

18. Lerum TV, Aaløkken TM, Brønstad E, et al. Dyspnoea, lung function and CT findings three months after hospital admission for COVID-19. *Eur Respir J*. Published online December 10, 2020. doi:10.1183/13993003.03448-2020\

19. Liang L, Yang B, Jiang N, et al. Three-month Follow-up Study of Survivors of Coronavirus Disease 2019 after Discharge. *J Korean Med Sci*. 2020;35(47):e418. doi:10.3346/jkms.2020.35.e418

© 2021 Nasserie T et al. *JAMA Network Open.*

20. Lu Y, Li X, Geng D, et al. Cerebral Micro-Structural Changes in COVID-19 Patients - An MRI-based 3-month Follow-up Study. *EClinicalMedicine*. 2020;25:100484. doi:10.1016/j.eclinm.2020.100484

21. Mandal S, Barnett J, Brill SE, et al. 'Long-COVID': a cross-sectional study of persisting symptoms, biomarker and imaging abnormalities following hospitalisation for COVID-19. *Thorax*. Published online November 10, 2020. doi:10.1136/thoraxjnl-2020-215818

22. Mazza MG, Palladini M, De Lorenzo R, et al. Persistent psychopathology and neurocognitive impairment in COVID-19 survivors: effect of inflammatory biomarkers at three-month follow-up. *Brain Behav Immun*. Published online February 24, 2021. doi:10.1016/j.bbi.2021.02.021

23. Méndez R, Balanzá-Martínez V, Luperdi SC, et al. Short-term neuropsychiatric outcomes and quality of life in COVID-19 survivors. *J Intern Med*. Published online February 3, 2021. doi:10.1111/joim.13262

24. Moreno-Pérez O, Merino E, Leon-Ramirez J-M, et al. Post-acute COVID-19 Syndrome. Incidence and risk factors: a Mediterranean cohort study. *J Infect*. Published online January 12, 2021. doi:10.1016/j.jinf.2021.01.004

25. Munro KJ, Uus K, Almufarrij I, Chaudhuri N, Yioe V. Persistent self-reported changes in hearing and tinnitus in post-hospitalisation COVID-19 cases. *Int J Audiol*. 2020;59(12):889-890. doi:10.1080/14992027.2020.1798519

26. Nguyen NN, Hoang VT, Lagier J-C, Raoult D, Gautret P. Long-term persistence of olfactory and gustatory disorders in COVID-19 patients. *Clin Microbiol Infect Off Publ Eur Soc Clin Microbiol Infect Dis*. Published online January 5, 2021. doi:10.1016/j.cmi.2020.12.021

27. Poncet-Megemont L, Paris P, Tronchere A, et al. High Prevalence of Headaches During Covid-19 Infection: A Retrospective Cohort Study. *Headache*. Published online August 5, 2020. doi:10.1111/head.13923

28. Puntmann VO, Carerj ML, Wieters I, et al. Outcomes of Cardiovascular Magnetic Resonance Imaging in Patients Recently Recovered From Coronavirus Disease 2019 (COVID-19). *JAMA Cardiol*. Published online July 27, 2020. doi:10.1001/jamacardio.2020.3557

29. Qu G, Zhen Q, Wang W, et al. Health related quality of life of COVID-19 patients after discharge: A multicenter follow up study. *J Clin Nurs*. Published online March 3, 2021. doi:10.1111/jocn.15733

30. Raman B, Cassar MP, Tunnicliffe EM, et al. Medium-term effects of SARS-CoV-2 infection on multiple vital organs, exercise capacity, cognition, quality of life and mental health, post-hospital discharge. *EClinicalMedicine*. 2021;31:100683. doi:10.1016/j.eclinm.2020.100683

31. Rosales-Castillo A, García de los Ríos C, Mediavilla García JD. Persistencia de manifestaciones clínicas tras la infección COVID-19: importancia del seguimiento. *Med Clin (Barc)*. 2021;156(1):35-36. doi:10.1016/j.medcli.2020.08.001

32. Shah AS, Wong AW, Hague CJ, et al. A prospective study of 12-week respiratory outcomes in COVID-19-related hospitalisations. *Thorax*. Published online December 3, 2020. doi:10.1136/thoraxjnl-2020-216308

33. Sonnweber T, Sahanic S, Pizzini A, et al. Cardiopulmonary recovery after COVID-19 - an observational prospective multi-center trial. *Eur Respir J*. Published online December 10, 2020. doi:10.1183/13993003.03481-2020

34. Sonnweber T, Boehm A, Sahanic S, et al. Persisting alterations of iron homeostasis in COVID-19 are associated with non-resolving lung pathologies and poor patients' performance: a prospective observational cohort study. *Respir Res*. 2020;21(1):276. doi:10.1186/s12931-020-01546-2

35. Sykes DL, Holdsworth L, Jawad N, Gunasekera P, Morice AH, Crooks MG. Post-COVID-19 Symptom Burden: What is Long-COVID and How Should We Manage It? *Lung*. Published online February 11, 2021. doi:10.1007/s00408-021-00423-z

36. Taboada M, Moreno E, Cariñena A, et al. Quality of life, functional status, and persistent symptoms after intensive care of COVID-19 patients. *Br J Anaesth*. 2021;126(3):e110-e113. doi:10.1016/j.bja.2020.12.007

37. Tomasoni D, Bai F, Castoldi R, et al. Anxiety and depression symptoms after virological clearance of COVID-19: A cross-sectional study in Milan, Italy. *J Med Virol*. Published online August 25, 2020. doi:10.1002/jmv.26459

38. Townsend L, Dyer AH, Jones K, et al. Persistent fatigue following SARS-CoV-2 infection is common and independent of severity of initial infection. *PloS One*. 2020;15(11):e0240784. doi:10.1371/journal.pone.0240784

39. Ugurlu BN, Akdogan O, Yilmaz YA, et al. Quantitative evaluation and progress of olfactory dysfunction in COVID-19. *Eur Arch Oto-Rhino-Laryngol Off J Eur Fed Oto-Rhino-Laryngol Soc EUFOS Affil Ger Soc Oto-Rhino-Laryngol - Head Neck Surg*. Published online January 1, 2021. doi:10.1007/s00405-020-06516-4

40. Vaira LA, Hopkins C, Petrocelli M, et al. Smell and taste recovery in coronavirus disease 2019 patients: a 60-day objective and prospective study. *J Laryngol Otol*. 2020;134(8):703-709. doi:10.1017/S0022215120001826

© 2021 Nasserie T et al. *JAMA Network Open*.

41. van den Borst B, Peters JB, Brink M, et al. Comprehensive health assessment three months after recovery from acute COVID-19. *Clin Infect Dis Off Publ Infect Dis Soc Am*. Published online November 21, 2020. doi:10.1093/cid/ciaa1750

42. Weerahandi H, Hochman KA, Simon E, et al. Post-Discharge Health Status and Symptoms in Patients with Severe COVID-19. *J Gen Intern Med*. Published online January 14, 2021. doi:10.1007/s11606-020-06338-4

43. Wong AW, Shah AS, Johnston JC, Carlsten C, Ryerson CJ. Patient-reported outcome measures after COVID-19: a prospective cohort study. *Eur Respir J*. Published online October 2, 2020. doi:10.1183/13993003.03276-2020

44. Xiong Q, Xu M, Li J, et al. Clinical sequelae of COVID-19 survivors in Wuhan, China: a single-centre longitudinal study. *Clin Microbiol Infect Off Publ Eur Soc Clin Microbiol Infect Dis*. 2021;27(1):89-95. doi:10.1016/j.cmi.2020.09.023

45. Zhao Y-M, Shang Y-M, Song W-B, et al. Follow-up study of the pulmonary function and related physiological characteristics of COVID-19 survivors three months after recovery. *EClinicalMedicine*. 2020;25:100463. doi:10.1016/j.eclinm.2020.100463
